# Hook Proteins: Association with Alzheimer Pathology and Regulatory Role of Hook3 in Amyloid Beta Generation

**DOI:** 10.1371/journal.pone.0119423

**Published:** 2015-03-23

**Authors:** Lydia Herrmann, Caspar Wiegmann, Annika Arsalan-Werner, Isabel Hilbrich, Carsten Jäger, Katharina Flach, Anne Suttkus, Ingolf Lachmann, Thomas Arendt, Max Holzer

**Affiliations:** 1 Paul Flechsig Institute of Brain Research, Department of Molecular and Cellular Mechanism of Neurodegeneration, University of Leipzig, Leipzig, Germany; 2 AJ Roboscreen GmbH, Leipzig, Germany; National Center for Geriatrics and Gerontology, JAPAN

## Abstract

Defects in intracellular transport are implicated in the pathogenesis of Alzheimer’s disease (AD). Hook proteins are a family of cytoplasmic linker proteins that participate in endosomal transport. In this study we show that Hook1 and Hook3 are expressed in neurons while Hook2 is predominantly expressed in astrocytes. Furthermore, Hook proteins are associated with pathological hallmarks in AD; Hook1 and Hook3 are localized to tau aggregates and Hook2 to glial components within amyloid plaques. Additionally, the expression of Hook3 is reduced in AD. Modelling of Hook3 deficiency in cultured cells leads to slowing of endosomal transport and increases β-amyloid production. We propose that Hook3 plays a role in pathogenic events exacerbating AD.

## Introduction

Alzheimer’s disease (AD) is a dementing disorder associated with two pathological hallmarks, extracellular amyloid plaques containing aggregated β-amyloid and intracellular deposits composed of hyperphosphorylated tau protein. Previous studies have shown that the pathology of AD is accompanied by defects in intracellular trafficking, associated with a decrease in axonal microtubules, a retardation of microtubule-based transport that leads to accumulation of cargo in axonal swellings and a dysfunction of the endosomal-lysosomal system [1–7]. In brain regions susceptible to tau pathology an enlargement and an increased number of endosomes are observed early in the pathogenesis [6,8,9]. Endosomal enlargement is accompanied by enhanced expression of the regulatory GTPases rab5 and rab7, which regulate trafficking of early and late endosomes [10,11]. Upregulation of the endocytic pathway precedes enhanced endocytosis of amyloid precursor protein (APP) and β-amyloid production [9,12]. In cell culture models this phenotype can be provoked by expression of rab5, amyloid precursor protein (APP) or the APP C-terminal fragment generated by beta-site APP-cleaving enzyme 1 (BACE1), by inhibition of dynein-dependent transport and by inhibition of endosome-lysosome fusion [13,14,15]. The cause and dynamics of endosomal dysfunction *in vivo* in AD, however, is poorly understood.

We propose that Hook proteins might be associated with both, AD pathology and endosomal transport. Hook proteins are a family of cytoplasmic linker proteins with a conserved N-terminal microtubule binding domain, a central coiled-coil region and a C-terminal domain functioning in protein and organelle binding [16]. Human Hook3 anchors the Golgi apparatus to microtubules and Hook2 is localized at the centrosome via its interaction of the C-terminal domain with centrosomal proteins [16,17,18]. Additionally, Hook proteins are implicated in endosomal transport. The Hook ortholog in *D*. *melanogaster* alters the trafficking of transmembrane receptor ligands and negatively regulates the fusion of late endosomes with lysosomes [19–22]. In humans all three Hook proteins are associated in a protein complex that interacts with the homotypic vesicular protein sorting (HOPS) complex to facilitate endosomal trafficking to lysosomes [23]. Furthermore, Hook2 is implicated in formation of aggresomes [24]. Aggresome formation is important in neurons for degradation of tau aggregates in AD. In *C*. *elegans* mutation of the Hook interacting partner SUT-2 suppresses tau aggregation and neurodegeneration [25]. Mammalian SUT-2 interacts with human Hook2 and its knockdown decreases susceptibility of mammalian cells to tau toxicity [25,26].

To explore the role of Hook proteins in neurons, especially in AD, we analyzed Hook protein expression in human brain tissue of controls, AD patients and in P301L-tau transgenic mice. We found that Hook2 is localized to glial cells. Therefore a direct neuroprotective function of Hook2 through facilitation of aggresome formation is improbable. Furthermore, Hook1 and Hook3 are expressed in neurons and relocate from the cytoplasm to neurofibrils in AD. Because Hook3 is specifically depleted in AD, we studied the effects of decreased Hook3 availability on endosomal transport and β-amyloid production in mammalian cell culture. We found that downregulation of Hook3 expression slows down endosomal transport and increases β-amyloid production. Thus we propose that Hook3 might play a critical role in pathogenic events exacerbating AD.

## Methods

### Antibodies

Polyclonal antibodies against Hook1, Hook2, Hook3 and pan-Hook were raised in rabbits using standard protocol (Biogenes) by injecting KLH-conjugated C-terminal peptides of each Hook protein: RRNLSVKVPAATSD (Hook1), RLASLNLRPTDKH (Hook2) and RRSYPGHVQPATAR (Hook3). The peptide QLEQLQEENFRLEC presenting a conserved sequence from the coiled coil domain was used to raise a pan-Hook antiserum. Hook-specific antibodies were affinity-purified using peptides coupled to CNBr-activated agarose (Hook1-Hook3 peptide) or epoxy-activated sepharose (pan-Hook peptide). Specificity of obtained Hook isoform-specific antisera was tested by Western blotting against all Hook isoforms expressed in N2A cells and against paired helical filaments (PHF) prepared as described by Greenberg and Davies [27]. Biotinylation was carried out with 1 mg of Hook antibodies and 80 μg biotinamido caproate N-hydroxysuccinimide ester (Sigma) in 1 ml of 0.1 M NaHCO_3_ buffer, pH 8.5. Following 4 h of incubation 0.2 M glycine was added. The antibodies were purified with NAP-10 columns (GE Healthcare) and equilibrated with PBS, pH 7.4.

Additionally, we used mouse antibodies against dynein intermediate chain (DIC): 74–1 (Santa Cruz), α-tubulin (DM1A), β-actin (AC-74) and S100β (SH-B1, all Sigma), p150 and p50 (BD Transduction Laboratories), pan-tau (clone 8F10, Analytik Jena), phospho-tau (AT8 and AT100, Thermo Scientific), APP (clone 22C11 Chemicon), β-amyloid (6F/3D, Dako) and rabbit antibodies against kinesin light chain 1 (KLC1, Santa Cruz), beta-site APP-cleaving enzyme 1 (BACE1, Calbiochem), ionized calcium binding adaptor molecule 1 (IBA1, Wako Chemicals), glial fibrillary acidic protein (GFAP, Dako), pan-tau (A0024, Dako) and goat anti-Presenilin 1 (PS1 N-19, Santa Cruz).

### Case recruitment and brain tissue preparation

Brains from 16 patients with AD (mean age ± SD, 80.3 ± 7.6 years) and 8 healthy controls dying without any history of neurological illness (76.8 ± 8.1 years) were used (Table 1). Each case of AD met the National Institute of Neurologic and Communicative Disorders and Stroke criteria for definite diagnosis of AD. All procedures of acquisition of the patient's personal data, autopsy and the handling of the autopsy material have been approved by the Ethical Committee of Leipzig University (License# 063/2000). Written informed consent from the donor or next of kin was obtained for use of human brain samples. Severity of AD pathology was scored according to Braak and Braak [28]. Six patients were classified as stages I-III (early stages of the disease) and 10 cases as stages V-VI (advanced stages of the disease). The brains were cut into 15-mm slabs. One hemisphere was fixed in 4% phosphate-buffered paraformaldehyde (PFA), pH 7.4 for 9 days and cryoprotected in 30% sucrose. The other hemisphere was snap frozen and stored at-80°C. Probes of 500 mg of the temporal cortex (Brodmann area 22) were homogenized with a Ultra Turrax disperser (IKA) in 3.75 ml of homogenization buffer (PBS, complete protease inhibitor (Roche), 1 mM NaVO_4_, 1 mM EDTA, 25 mM NaF, 2 mM EGTA, 10 mM β-glycerophosphate, 1 mM MgCl_2,_ 2 μg/ml leupeptin, 0.5 mM calpain inhibitor I) on ice. Subsets of the homogenate was supplemented with 10% glycerol (Sigma) and stored at-80°C (whole homogenate for ELISA analysis). The remaining part was ultracentrifuged at 110000 *g* for 1 h at 4°C in a T865 rotor (Sorvall Combi Plus ultra centrifuge). The supernatant was collected, supplemented with 10% glycerol (Sigma) and stored at-80°C (cytoplasmic fraction for Western blot analysis). Protein concentrations were determined using Bradford Reagent (Thermo Scientific). Of each tissue sample 23 μg of protein were boiled with SDS sample buffer (125 mM Tris-HCl pH 6.8, 4% SDS, 20% glycerol, 10% β-mercaptoethanol, 0.02% bromphenol blue) for 5 min, analyzed by 9% sodium dodecyl sulphate-polyacrylamide gel electrophoresis (SDS-PAGE). Via densitometric analysis using TINA software the intensities of the bands were determined. An exact Mann-Whitney U test was carried out with SPSS 15 software to compare protein levels between different groups.

**Table 1:** Case details.

Case number	AD stage (Braak)	Gender	Age (years)	Cause of death	Postmortem delay (h)
1	0	m	66	Haemorragical shock	24
4	0	f	82	Diabetes, hypertension	30
5	0	m	64	Cardiovascular failure	30
12	0	f	79	Renal insufficiency	33
15	0	m	82	Cardiovascular failure	46
16	0	f	84	Cardiovascular failure	12
20	0	f	84	Cardiovascular failure	36
22	0	m	73	Cardiovascular failure	24
Mean ± SD			80.3 ± 7.6		29.4 ± 10
2	I-II	f	60	Pneumonia, hypertension	26
6	I-II	f	81	Cardiovascular failure	58
9	III	m	84	Liver failure	29
10	I-II	f	73	Cardiovascular failure	34
14	I-II	m	75	Pneumonia	48
17	I-II	m	76	Cardiovascular failure	46
Mean ± SD			74.8 ± 8.3		40.2 ± 12.5
3	V-VI	f	77	Gastrointestinal bleeding	34
7	V-VI	f	82	Cardiovascular failure	48
8	V-VI	f	88	Pulmonary embolism	48
11	V-VI	f	86	Cardiac insufficiency	40
13	V-VI	f	92	Pneumonia	48
18	V-VI	f	83	Cardiovascular failure	72
19	V-VI	m	75	Pneumonia	48
21	V-VI	f	82	Cardiovascular failure	24
23	V-VI	m	85	Cardiovascular failure	48
24	V-VI	f	86	Pulmonary embolism	54
Mean ± SD			83.6 ± 5		46.4 ± 12.6

#### Mus musculus

All experimental procedures on animals were carried out in accordance with the European Council Directive of November 24th 1986 (86/609/EEC) and all efforts were made to minimize suffering. This study has been approved by the ethics committee (T09/09, Veterinärwesen, Landesdirektion Sachsen). For murine brain tissue preparation we used 6 month old P301L-tau transgenic mice (Taconic stock# 002508-M) [29] which are back crossed into a mouse-tau null background (The Jackson Laboratory stock# 004779) [30]. The mice were killed by CO_2_-asphyxia, perfused with isotonic saline solution containing 0.01% heparin 25000 (Ratiopharm) and subsequently with 4% PFA, 0.1% glutaraldehyde. The brain tissue was postfixed in 4% PFA, 0.1% glutaraldehyde for 24 h and conserved in 30% sucrose at 4°C. Tissue processing was performed as described [31].

### Western blotting

Proteins separated by SDS-PAGE were transferred onto polyvinyliden fluoride (PVDF) membrane (Polyscreen, DuPont) overnight using a tank blot system (TV400, biostep). Membranes were incubated in TBS, 0.1% Tween-20 (Sigma) containing 2% bovine serum albumin (BSA, Roth) and probed with antibodies for 2 h at room temperature. After washing with TBS, 0.1% Tween-20, blots were immunoreacted with horseradish peroxidase (HRP) conjugated donkey anti-rabbit and goat anti-mouse antibodies (GE Healthcare). Chemoluminescence was visualized on a Kodak Image Station 2000R using ECL substrate (Lumigen TMA-6, GE Healthcare)

### ELISA

Microtitration plates were coated with Hook isoform-specific antibodies 5 μg/ml in TBS, 0.5 mM EDTA (Roth) and blocked with 0.5% cold fish gelatine (Sigma). Brain homogenates were diluted 1:5 with homogenization buffer and 50 μl were incubated for 90 min. After washing, the wells were probed with biotinylated pan-Hook antibody for 2 h or alternatively with AT100. In case of AT100 detection the wells were subsequently incubated with biotinylated sheep anti-mouse IgG antibody (GE Healthcare). Then the wells were washed and HRP conjugated avidin (ExtrAvidin, Sigma) was added. Color development was carried out with 100 μl 0.4 mg/ml orthophenylene diamine (Sigma) in acetate buffer (0.1 M sodium acetate, pH 5), stopped with 50 μl 1 M HCl and optical density was read at 490 nm. Positive controls of Hook protein containing cell lysates and negative controls without lysate, capture or detection antibody were included in each assay. All probes were determined in quadruplet. Mean of data were corrected by the negative control which was not incubated with any homogenate and statistics were performed as described above. For the Hook3-Tau binding assay recombinant Hook3 was expressed in BL21-CodonPlus(DE3)-RIL transformed with pGex-Hook3. Hook3 comprised within inclusion bodies was extracted with urea buffer (7 M urea, 2 M thiourea, 1 mM DTT) for 20 min and centrifuged at 20000 g for 20 min at room temperature. GST-Hook3 was dialysed against 3 M urea, 0.4 M Na_2_SO_4_, 0.01% Tween-20 for 3 h and refolding was accomplished by continuous dilution of dialysis buffer with refolding buffer (50 mM Tris/HCl pH 7.4, 0.4 M Na_2_SO_4_, 150 mM NaCl, 0.1 mM DTT, 0.02% Tween-20) at 1 ml/min for 24 h. Soluble Hook3 was obtained after a final dialysis against refolding buffer, centrifugation, overnight incubation with Prescission protease (GE Healthcare) and adsorption of the GST-tag to MagneGST-beads (Promega).

10μg Hook3 was combined with 100μl Sephacryl-S500 purified monomeric 2N4R tau (fraction 47, 0.15mg/ml) or aggregated 2N4R tau (fraction 21, 0.14mg/ml) [32] as unphosphorylated or MAPK13 (Invitrogen)-phosphorylated tau species and incubated for 60 min at room temperature.

The protein mix was diluted 1:100 in TBS, 0.1% Tween-20 containing 1% bovine serum albumin and applied to a microtiter plate previously coated with the monoclonal pan-tau antibody 8F10 at 5μg/ml. Incubations containing Hook3 or 2N4R-tau only served as controls. The amount of Hook3 captured by the anti-tau antibody was then detected using the polyclonal Hook3 antibody, HRP-conjugated anti-rabbit antibody and tetramethylbenzidine as peroxidase substrate. Hook3 immunoreactivity was corrected for background reactivity and equal binding of different tau species was verified by detection with the polyclonal tau antibody (Dako).

Quantification of secreted β-amyloid in culture media of N2A cells transfected with Hook siRNAs and incubated for 48 h was done with Mouse/Rat Amyloidβ (1–40) (N) Assay Kit (IBL, Hamburg) according to the manufacturer's instructions.

### Dot Blot

N2A cells expressing EGFP-Hook2 fusion protein were lysed in 6 M urea by sonication (Cell Disrupter, Virsonic). Subsequently, 100 ng of Hook2 cell lysate and 100 ng of β-amyloid were spotted onto two 0.2 μm nitrocellulose membranes. After blocking, the membrane was probed with anti-β-amyloid and anti-Hook2 antibodies (1 μg/ml each). Detection was performed as described for Western blotting.

### Cell culture and transfection

HeLa and N2A cells (Leibniz Institute DSMZ-German Collection of Microorganisms and Cell Cultures) were cultured at 37°C in DMEM/F-12 (Biochrom) supplemented with 5% fetal calf serum (FCS, Biochrom), 1% L-glutamine (GIBCO), 1% non-essential amino acids (Biochrom) and 1% penicillin/streptomycin (PAA). Alexa Fluor 488-labeled AllStars negative control siRNA, Hook3 siRNA1 (CAGCATGAGAATAAGATGTTA) and Hook3 siRNA2 (CTCAATCAATCTGATTCTATA), that efficiently downregulates Hook3 in human and murine N2A cells were synthesized by Qiagen. For transfection 25 ng siRNA and 2 μl HiPerFect reagent (Qiagen) were added to 100 μl OptiMEM (Gibco). After 10 min, 1.2 × 10^5^ cells were seeded in a 24 well, the siRNA was added followed by 4 h of incubation in DMEM/F-12 with 5% FCS for 3 days. Transfection rate of control siRNA was 90%. Transfection of pEGFP and pEGFP-Hook1–3, pEGFP-dynamin 1 K44A (dominant-negative dynamin 1, addgene plasmid 22197 [33]) and APP-YFP [34] vectors was carried out with Lipofectamine 2000 (Invitrogen). For use in SDS-PAGE, transfected cells were washed twice with PBS and scraped off the plate, pelleted and heated in SDS sample buffer.

### Sedimentation analysis

Transfected N2A cells grown in 94-mm dishes were harvested in PBS, 10 mM EDTA, pelleted and resuspended in 200 μl lysis buffer: buffer A (50 mM Tris, pH 7.5, complete protease inhibitor, 150 mM NaCl, 1 mM EDTA, 0.5 mM AEBSF, 1 μg/ml PepstatinA) supplemented with 0.2% NP-40 (Fluka). Lysates were kept on ice for 30 min and sonicated for 20 sec. Following 15 min of centrifugation at 15000 *g* the supernatant was laid on top of a 3 ml 5–20% sucrose gradient in buffer A. For each experiment sedimentation standards thyroglobulin (19S), katalase (13S) and BSA (3S) were run separately. The gradients were centrifuged in an AH650 rotor (Sorvall Combi Plus) at 65000 *g* for 18 h at 4°C. Subsequently, 200 μl fractions were collected and equal volumes of all fractions were analyzed by immunoblotting.

### EGF Uptake

HeLa cells grown on coverslips were transfected with siRNA. After pre-incubation in serumfree DMEM for 1 h, cells were treated with 100 ng/ml Alexa Fluor 555-EGF (Molecular Probes) at 37°C for the indicated periods. Cells were washed twice with 0.1 M acetate buffer, pH 5 and fixed for 20 min with 4% PFA containing 3% sucrose. Ethanol dehydrated cells were embedded in Entellan (Merck). Localization of endosomes was observed using a confocal microscope LSM 510 (Carl Zeiss). Image analysis was performed blinded by characterization of intracellular distribution of Alexa Fluor 555-EGF using ImageJ (NIH, version 1.45s). An EGF-fluorescence intensity profile across each cell was calculated and the intensity difference of fluorescence maximum and minimum averaged over a 1.5 μm section was used to characterize perinuclear EGF-fluorescence clustering.

### Immunohistochemistry

The temporal (Brodmann area 22), the frontal (Brodmann area 10), the occipital cortex (Brodmann area 17) and the hippocampus (Brodmann area 38) of 5 AD brains at Braak stages II-VI (77.6 ± 5 years) and of 5 control brains (age 73.6 ± 5.4 years) were processed as described [35]. Briefly, the tissue was cut into 30 μ slices and endogenous peroxidase activity was quenched in 1% H_2_O_2_. The tissue was microwaved for 45 sec in 10 mM citrate buffer, pH 6 to enhance antigen retrieval and non-specific binding sites were masked by 1 h of incubation in a blocking solution containing 2% BSA, 1% normal goat serum and 0.01% Triton X-100 in TBS. Free-floating sections were incubated overnight (4°C) with 1 μg/ml anti-Hook antibodies. Immunoreactivity was visualised using the biotin-avidin-system (biotinylated goat anti-rabbit IgG, HRP-conjugated avidin) and 3,3’-diaminobenzidine (Sigma) as chromogen. For immunofluorescence, the H_2_O_2_ quenching step was omitted and detection was performed with Cy5-labeled goat anti-mouse and Cy2-labeled goat anti-rabbit antibodies (Jackson Immunoresearch). Biotinylated Hook2 antibody was used for double labeling when the second primary antibody was also raised in rabbit (anti-IBA1, anti-GFAP). Tissue autofluorescence was quenched by incubation with 1% Sudan Black B in 70% ethanol before coverslipping.

### RNA isolation and quantitative real-time PCR (qRT-PCR)

Total RNA was extracted from brain tissue (Brodmann area 22) with Trizol reagent (Life Technologies) and the quality of the isolated RNA was controlled with denaturing RNA electrophoresis. RNA with defined 28S and 18S rRNA bands was further purified with Qiagen RNeasy columns according to the manufacturer's instructions (Qiagen). For reverse transcription a mix of oligodT primers/ random hexamer primers and SuperscriptIII-Enzyme (Invitrogen) were used. Quantitative RT-PCR was performed using 5 μl 1:10 diluted cDNA with 15 μl of a HotStarTaq-Plus mastermix (Qiagen) containing forward and reverse primers and Taqman probes (see below). Amplifications were carried out on a RotorGene 6000 (Qiagen, Hilden) at 96°C for 5 min, followed by 40 cycles of 95°C for 30 s, 56°C for 30 s, and 72°C for 45 s. A qRT-PCR for MAPT was performed in parallel for normalization purposes. For calculation of total mRNA molecule numbers a standard curve was created for each primer pair by amplification of diluted Hook isoform plasmid-cDNA (tenfold serial dilution). PCR-specific products were determined as a single peak at the melting curves. Primer specificity was also confirmed by agarose gel electrophoresis of the qRT-PCR products. The average threshold cycle value (Ct) was calculated from three replicates of each cDNA sample. For RT-qPCR the following primers and probes were used:

Hook1 forward primer: 5’-AACAGGACCACCTAAACCAAACAG-3’

Hook1 reverse primer: 5’-TTCTAGCTGCTCCTGAAGTTCC-3’

Hook1 TaqMan probe: 5’FAM-CTTCGCTTACAGCAAGAAGGCTCTGAGAA-BHQ1–3’,

Hook2 forward primer: 5’-CCTGAAGGATGAGATGGATGAAC-3’

Hook2 reverse primer: 5’-AGCTCATCCTCCAGTTGTCGC-3’

Hook2 TaqMan probe: 5’-FAM-CCGGCGTTGCGTTCCTCCAG-BHQ1–3’,

Hook3 forward primer: 5’-TTCGGCAACAGAATGATGAAC-3’

Hook3 reverse primer: 5’-AGTTAAGAAAGGCCAACGCAG-3’

Hook3 TaqMan probe: 5’-FAM-AACCTGCCGCCTTAAATCACCAAGG-BHQ1–3’,

MAPT forward primer: 5’-CCGCCAGGAGTTCGAAGTGA-3’

MAPT reverse primer: 5’-AGCTTCGTCTTCCAGGCTGG-3’

MAPT TaqMan probe: 5’-FAM-CCAACCCGTACGTCCCAGCGTGGATC-BHQ-1–3’

The use of MAPT as a reference gene was founded on the notion that MAPT mRNA has stable expression during progression of AD as shown by several methods [36,37,38]. Alternative splicing of Hook isoforms may confound qRT-PCR measurements due to absence of exons necessary for primer or Taqman probe binding. However, no alternative splicing of exons has been found in five controls and five AD patients, in fetal human brain and Hela cells. (data not shown).

## Results

### Hook proteins are associated with pathological hallmarks in AD brain

**Fig 1:**
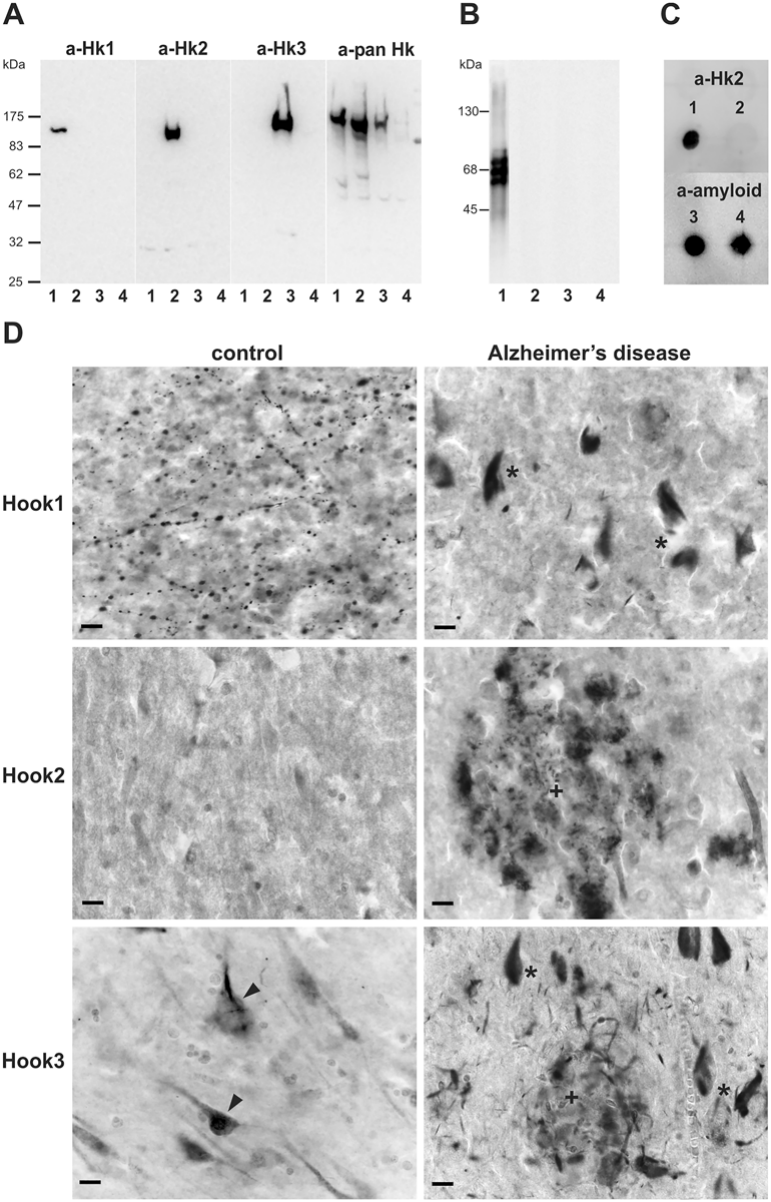
High specificity of Hook isoform-specific antibodies and Hook isoform localization in control brain and AD brain. (a) Affinity purified Hook isoform-specific antibodies were tested on lysates of N2A cells transfected with pEGFP-Hook plasmids. Lysates of N2A cells expressing EGFP-Hook1 (lane 1), EGFP-Hook2 (lane 2), EGFP-Hook3 (lane 3) and EGFP only (lane 4) were separated on 10% SDS-PAGE and transferred to PVDF membrane. The membrane was probed with 0.5 μg/ml anti-Hook1 antiserum (a-Hk1), 0.5 μg/ml anti-Hook2 antiserum (a-Hk2), 0.5 μg/ml anti-Hook3 antiserum (a-Hk3) or 0.25 μg/ml pan-Hook antiserum (a-pan Hk). Pan-Hook antiserum shows the expression of all EGFP-Hook fusion proteins in N2A cells (100–120 kDa), whereas the isoform-specific antisera only label the corresponding isoform. (b) No crossreactivity of affinity-purified Hook antibodies with PHF-tau prepared from AD brain was detected, phospho-tau antibody AT8 (lane1), a-Hook1 (lane 2), a-Hook2 (lane 3), a-Hook3 (lane 4). (c) The Dot blot shows that Hook2 antibody does not crossreact with β-amyloid; 100 ng EGFP-Hook2 N2A cell lysate (spot 1, 3); 100 ng β-amyloid 1–40 (spot 2, 4). (d) Immunohistochemical staining of the hippocampal CA3 region using Hook1, Hook2 and Hook3 antiserum in control brain and in AD brain (Braak stage V). Hook1 and Hook3 antibodies label tau pathology such as neurofibrillary tangles (asterisk) and dystrophic neurites in neuritic plaques (+). Granular neuronal Hook3 immunoreactivity present in control brains (arrowhead) is reduced in AD and NFT-associated Hook3 immunoreactivity is predominant. Amyloid plaque staining (+) is observed using the Hook2 isoform-specific antibody. Scale bar = 20 μm.

To analyze the distribution of Hook proteins in control and AD brain, we prepared affinity-purified rabbit antibodies directed against a C-terminal epitope, where sufficient sequence diversity between Hook isoforms occurs. In addition, we raised a pan-Hook antibody against an epitope in the coiled-coil region, which is conserved between the Hook isoforms, but not present in the Hook-like proteins. The specificities of these polyclonal antibodies were verified by cross-testing each antiserum against all Hook isoforms by Western blotting (Fig. 1a) and immunofluorescence staining (data not shown) of EGFP-Hook-transfected cell lines. Fig. 1a shows the isoform-specificity of each Hook antiserum and the reactivity of the pan-Hook antibody with all three isoforms on Western blots. Furthermore, to rule out cross-reaction of Hook antibodies with aggregated tau and β-myloid, we prepared soluble paired helical filament (PHF)-tau. No reactivity of Hook antibodies towards PHF-tau was observed on Western blots (Fig. 1b). In addition, Hook2 antiserum failed to detect β-amyloid peptides on Dot blot (Fig. 1c).

**Fig 2:**
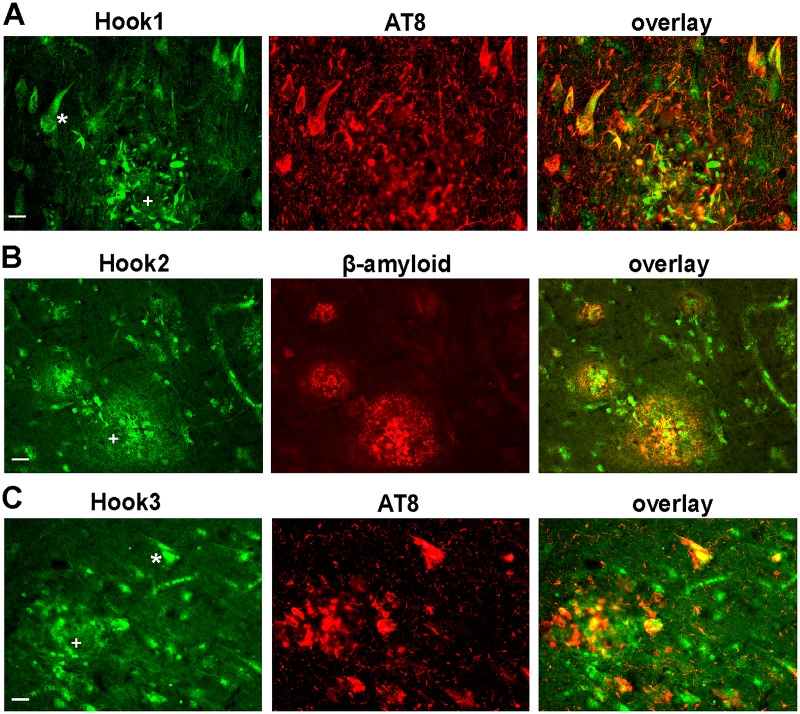
Localization of Hook1, Hook2 and Hook3 in relation to pathological markers in AD brain. (a) Hook1 detection in the hippocampus reveals strong labeling of neurofibrillary tangles (asterisk) and dystrophic neurites of neuritic plaques (+) co-labeled by AT8. Dystrophic neurites are detected more clearly by anti-Hook1 compared to AT8. (b) Hook2 protein is enriched in cellular components within amyloid plaques detected by anti-amyloid antibody 6F3D (+), but not overlapping with β-amyloid localization. Hook2 is not associated with tau aggregates. (c) Hook3 is expressed in neurons and labeling is increased in tangle-bearing neurons (asterisk) and dystrophic neurites (+) detected with AT8. Scale bar = 20 μm.

Next, we analyzed the expression and localization of each Hook isoform in the hippocampus (Fig. 1d, S1 Fig.) and in the temporal, the frontal and the occipital cortex (S2–S4 Figs.) of 5 control and 5 AD brains. Immunohistochemistry of human control brain tissue reveals a unique staining for each Hook antiserum. In addition to the parenchymal staining, Hook1 antibody labels axonal varicosities in the cortex of human controls (Fig. 1d, S2 Fig.). The parenchymal staining is reduced in AD brain and neurofibrillary tangles or dystrophic neurites are strongly labeled. In controls Hook2 antiserum reacts weakly with blood vessels, nuclei of small cells and occasionally with GFAP-positive astrocytes with stellate morphology (Fig. 1d, S3 Fig., and S5 Fig.). A plaque-like Hook2-staining is observed in AD brain, which depicts certain structures inside amyloid plaques (Fig. 2b, S5 Fig.). It presents a staining of cellular components, which can be assigned to astrocytic extensions immunoreactive to S100β.

A strong ubiquitous neuronal staining in all subfields of the hippocampus and the cortical areas was observed with the Hook3 antiserum as depicted in Fig. 1d, S1 Fig. and S4 Fig. More intense immunoreactivity was found in the cytoplasmic and nuclear compartment of pyramidal cells of layer III and layer V in the temporal cortex. The cytoplasmic Hook3 immunoreactivity extends into the apical dendrites of pyramidal cells. In AD brain tissue the neuronal cytoplasmic staining by the Hook3 antiserum is diminished in most neurons. Additionally, neurofibrillary tangles as well as dystrophic neurites of senile plaques are labeled, similar to Hook1 staining. The intracellular colocalization of Hook1 or Hook3 with tau aggregates, detected with the phosphorylation-dependent tau antibody AT8, has been confirmed by immunofluorescence double-labeling and confocal laserscanning microscopy (Fig. 2a and c). In summary, all three isoform-specific Hook antisera stain pathological hallmarks in AD brain.

### Confirmation of Hook-tau colocalization in a mouse model of tau pathology

**Fig 3:**
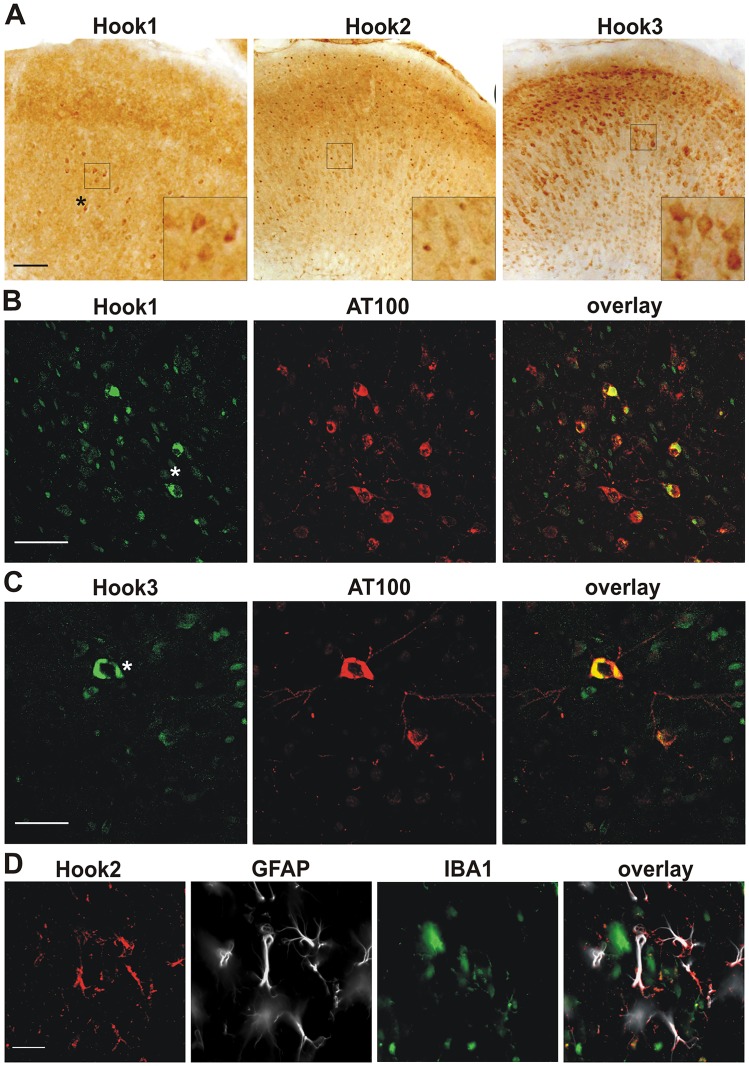
Immunohistochemical staining of Hook proteins in brain tissue of 6 month old P301L- tau transgenic mice. (a) Overview of Hook immunoreactivities in the prefrontal cortex (lateral of the central sulcus). Hook1 antibody shows a weak cytoplasmic immunoreactivity in the cortex, while Hook2 antibody marks distinct punctuate structures. Hook3 antibody clearly labels the soma of neurons. The staining shows a granular pattern. Note the tangle-like structures in the anti-Hook1 stained section (asterisk) (b) Staining of the brainstem with Hook1 and AT100 antibodies; tau-aggregates co-localise with Hook1. (c) Staining of the brainstem with Hook3 and AT100 antibodies. Hook3 antibody marks a neuron containing aggregated tau. (d) Staining of Hook2, GFAP and IBA1 in the brainstem; Hook2 co-localises with GFAP, but not with IBA1. Scale bars = a: 100 μm, b-d: 50 μm.

To analyze the distribution of Hook proteins in a mouse model with tau pathology, we conducted immunostaining of all three Hook proteins in tissue of P301L-tau transgenic mice. Hook1 antibody shows a diffuse staining pattern in the cortex whereas neurofibrillary tangle-like structures are strongly labeled (Fig. 3a). Like in human brain tissue, the strongest immunoreactivity was detected in Hook3-stained sections, where cell bodies and proximal dendrites and axons are visible. By testing for co-labeling of Hook1 and Hook3 with phospho-tau, a clear colocalization was observed (Fig. 3b and c). In the brain stem most Hook1 positive cells are also labeled by the AT100 antibody (85%), while only 35% of AT-100 labeled cells are Hook1-positive (Fig. 3b). Colocalization of Hook3 and phospho-tau is difficult to estimate, since the Hook3 antibody stains neuronal cells throughout the CNS. The immunoreactivity pattern of Hook3 positive cells is different in cells showing tangle-like tau-aggregates with AT100 labeling (Fig. 3c). It strongly resembles the tangle-like staining of the AT100 antibody.

In the Hook2 labeled sections we observed a punctuate staining pattern in the grey substance (Fig. 3a). In the white matter we saw small appendage-rich cells stained by Hook2 antibody appearing to be glial cells. To confirm that Hook2 localizes to glial cells, we performed co-labeling of murine Hook2 with the astroglia-marker GFAP and IBA1, a marker for microglia (Fig. 3d). The co-labeling shows that Hook2 immunoreactivity is strong in GFAP-positive cells. The intracellular localization differs slightly as the Hook2 antibody has the tendency to rather stain the extremities of astrocytes. IBA1-positive cells are rarely labeled by Hook2 antibody. Thus Hook2 is expressed in astrocytes rather than in microglia.

### Hook protein expression is reduced in brain tissue of patients with AD

**Fig 4:**
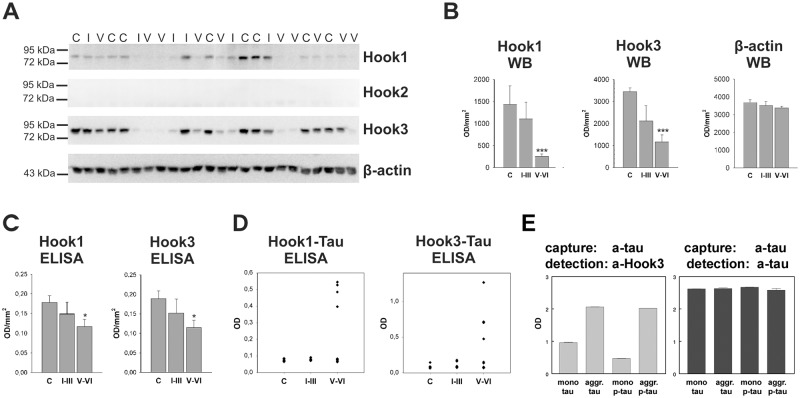
Hook protein levels in brain tissue of patients with AD. (a) Western blot analysis of the cytoplasmic fractions of brain homogenates. Grey substance of Brodmann area 22 (temporal cortex) was homogenized in detergent-free buffer and was subsequently ultracentrifuged. Twenty-three μg of protein of each supernatant were loaded on SDS-PAGE, analyzed by Western blot and probed with different antibodies. C: Braak stage 0, I: Braak stage I-III, V: Braak stage V-VI. (b) Densitometric analysis of protein immunoreactivity in brain homogenates of controls (Braak stage 0, n = 8), early stages of AD pathology (Braak stages I-III, n = 6) and late stages (Braak stages V-VI, n = 10). Data are means ± SEM. Statistical analysis was carried out with Kruskal-Wallis test and subsequently Mann-Whitney U test. * p < 0.05, *** p < 0.001 compared to control group with Mann-Whitney U test. OD: optical density, WB: Western blot. (c) Quantification of total Hook1 and total Hook3 in whole homogenates by ELISA. Whole homogenates comprising the soluble and the particulate fraction were prepared from tissue of Brodmann area 22 in detergent-free buffer. Hook proteins were captured from whole homogenates with isoform-specific Hook antibodies and detected with biotinylated pan-Hook antibody. Data were corrected by the negative control of the antibody background and groups were statistically tested with Kruskal-Wallis test and Mann-Whitney U test. Data are means ± SEM. (d) Association of Hook1 and Hook3 with PHF-tau analysed by a Sandwich ELISA. Hook proteins were captured from whole homogenates with anti-Hook1 or anti-Hook3 antibody. Detection with AT100 revealed an association of Hook1 and Hook3 with PHF-tau in four brain homogenates from patients with AD. (e) Hook3 binding to recombinant tau is increased by tau aggregation and not by tau phosphorylation. Recombinant Hook3 was incubated with 2N4R-tau either in its unmodified, aggregated (aggr.) or phosphorylated form (p-tau) or with both modifications and applied to a monoclonal pan-tau capturing antibody. Hook3 protein associated with captured tau protein was quantified using a polyclonal Hook3 antibody in this sandwich-ELISA (left panel). Hook3 immunoreactivity was corrected for background reactivity and equal binding of the different modified tau species was verified by detection with the polyclonal tau antibody (right panel).

To further investigate the expression of Hook proteins in AD, we used brain tissue from the temporal cortex (Brodmann area 22) staged according to Braak and Braak (Braak & Braak 1991) into three groups: controls, Braak stages I-III and stages V-VI. The cytoplasmic fractions of tissues were prepared by ultracentrifugation of grey substance from Brodmann area 22 homogenized in detergent-free PBS containing protease inhibitors. Western Blot analysis of the cytoplasmic fractions allowed us to quantify soluble Hook1 (85 kDa) and Hook3 (83 kDa, Fig. 4a). We were not able to detect any endogenous Hook2 immunoreactivity by Western blotting (for entire Western blots see S6 Fig.).

Densitometric analysis of Western blot immunoreactivity revealed a suggestive decrease of Hook1 and Hook3 in Braak stages I-III of AD and a significant decrease in Braak stages V-VI (Hook1 18% of control level, p = 9 x 10^-4^; Hook3 34% of control level, p = 5 x 10^-5^, Fig. 4b) compared to controls. In comparison, we found no significant change in the protein levels of β-actin (Fig. 4a and b). The amount of Hook1 varies in controls and in Braak stages I-III, whereas in Braak stages V-VI only a little amount of Hook1 was detected. In contrast, Hook3 is highly abundant in all control samples. In early stages of AD (Braak stages I-III) Hook3 expression is diminished in three out of six cases and a reduced Hook3 level was detected in all Braak stages V-VI samples (Fig. 4a).

Since the amount of Hook proteins contained within the particulate fraction is not amenable to quantification by SDS-PAGE due to poor solubility, we performed a sandwich-ELISA with tissue lysates prepared by homogenization of grey substance form Brodmann area 22. These whole homogenates comprise cytoplasmic and particle-bound proteins. We captured Hook1 and Hook3 from the whole homogenates with isoform-specific antibodies and detected the captured protein with pan-Hook antibody. Once again, we found a decrease in Hook proteins in samples from patients with AD with augmented severity of AD pathology (Fig. 4c). In the late stages of AD the reduction of Hook1 (66% of control level, p = 0.02) and Hook3 (61% of control level, p = 0.03) is less pronounced than in the cytoplasmic fractions. That leads to the assumption that Hook1 and Hook3 are partially retained at tau aggregates in the particulate fraction. To prove the association of Hook proteins with aggregated tau, we captured Hook1 and Hook3 from brain homogenates and detected with AT100, an antibody specific for PHF-tau. In four AD cases we found an association of Hook1 and Hook3 with PHF-tau (Fig. 4d). We further found no interaction of Hook proteins with non-pathological, soluble tau by coimmunoprecipitation and pull down assay (data not shown). Thus we suggest that binding of Hook1 and Hook3 to tau starts with its aggregation and posttranslational modifications in AD. Sequestration of Hook1 and Hook3 at tau aggregates additionally reduces the availability of those proteins in the cytoplasm.

However, the overall decrease in protein content is not related to a diminished Hook mRNA expression. qRT-PCR quantitation of Hook 1/3 mRNA revealed an unaltered expression levels in AD patients (S8 Fig.). Hook2 mRNA was significantly augmented in AD brain.

### Interaction of recombinant Hook3 with tau is dependent on tau aggregation

In order to discriminate, whether tau hyperphosphorylation or tau aggregation with concomitant beta-sheet secondary structure formation triggers Hook3-binding we used recombinant proteins to quantify this interaction using a sandwich ELISA. Unphosphorylated or MAPK13 phosphorylated recombinant 2N4R tau was subjected to heparin-induced aggregation for 48h and afterwards aggregated tau was separated from monomeric tau by size-exclusion chromatography. Monomeric tau and tau filaments in its unphosphorylated and hyperphosphorylated states were tested for Hook3 binding affinity (Fig. 4e). Aggregated tau species, regardless of their phosphorylation status, associate more strongly with Hook3.

### Reduced Hook3 expression slows down endosomal transport

**Fig 5:**
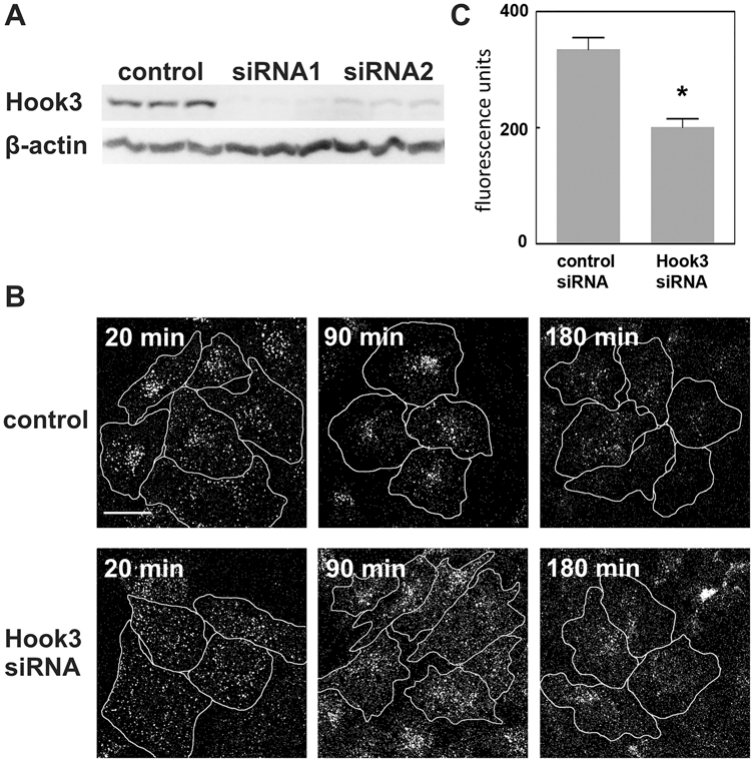
Effect of reduced Hook3 expression on endosomal transport and perinuclear clustering of EGF. HeLa cells were transfected with scrambled control RNA or one out of two Hook3 siRNAs and incubated for 72 h. (a) Knockdown of Hook protein levels. Cells were harvested 72 h after transfection and analyzed by SDS-PAGE and Western blotting. (b) Effect of Hook3 siRNA2 on endosomal trafficking of EGF. Cells were loaded with Alexa Fluor 555-EGF, incubated for the indicated intervals, fixed and analyzed by confocal microscopy. Hook3 siRNA slows down endosomal transport and perinuclear clustering of EGF indicated by less pronounced clustering of EGF-containing endosomes after 20 min and 90 min and an increased number of remaining fluorescent endosomes after 180 min of incubation. A similar result was obtained with Hook3 siRNA1. Scale bar = 20 μm. Cells are marked by cellular outlines. (c) Quantification of nuclear clustering by measuring the inhomogeneity of Alexa Fluor 555-EGF distribution inside the cell. Along a fluorescence intensity-profile a difference of fluorescence maximum and minimum was calculated. Mean and standard deviation are shown from four experiments (n = 4) using data from 15 cells each. Statistical significance was probed using Mann-Whitney U-Test * p< 0.05.

Hook3 is the most abundant Hook protein isoform in human brain tissue. Because of the reduction of Hook3 in all cytoplasmic fractions of tissue from the temporal cortex in late stages of AD (Fig. 4), we investigated the effect of a reduced availability of Hook3 in a cell culture model. Hook3 expression in HeLa cells transfected with control scrambled RNA oligonucleotides or one out of two Hook3 siRNAs was quantified by Western blot analysis (Fig. 5a). The expression of Hook3 is reduced by Hook3 siRNAs to 10% and 26% of control level, respectively. Transfected HeLa cells were incubated with fluorescence labeled EGF to study the effect of reduced Hook3 availability on endosomal transport. EGF is bound to the EGF-receptor and travels from early endosomes to late endosomes and afterwards to lysosomes, where it is degraded. HeLa cells transfected with control or Hook3 siRNA were loaded with Alexa Fluor 555-EGF. At different points in time, from 20 min up to 180 min, cells were fixed and analyzed by confocal microscopy (Fig. 5b). Endocytosis occurs in both, control and Hook3 siRNA transfected cells. The overall uptake of Alexa Fluor 555-EGF is not different between control siRNA (184.7 +/- 48.3 fluorescence units) compared to Hook3 siRNA (199.6 +/- 50.9 fluorescence units) showing that Hook3 siRNA is not affecting endocytosis of EGF (see quantification schematic S7 Fig.). However, the transport of EGF towards the cell center and the loss of EGF-fluorescence are delayed in Hook3 siRNA transfected cells indicated by a reduced endosomal clustering after 90 min and enhanced EGF fluorescence after 180 min of incubation compared to control cells. The slowing down of perinuclear clustering at 90 min after EGF application was characterized by a fluorescence difference calculated from a maximum fluorescence intensity region and a minimum fluorescence intensity region along a fluorescence profile (S7 Fig.). This analysis revealed a significant lack of perinuclear clustering in Hook3 siRNA transfected cells 90 min after EGF application (Fig. 5c).

**Fig 6:**
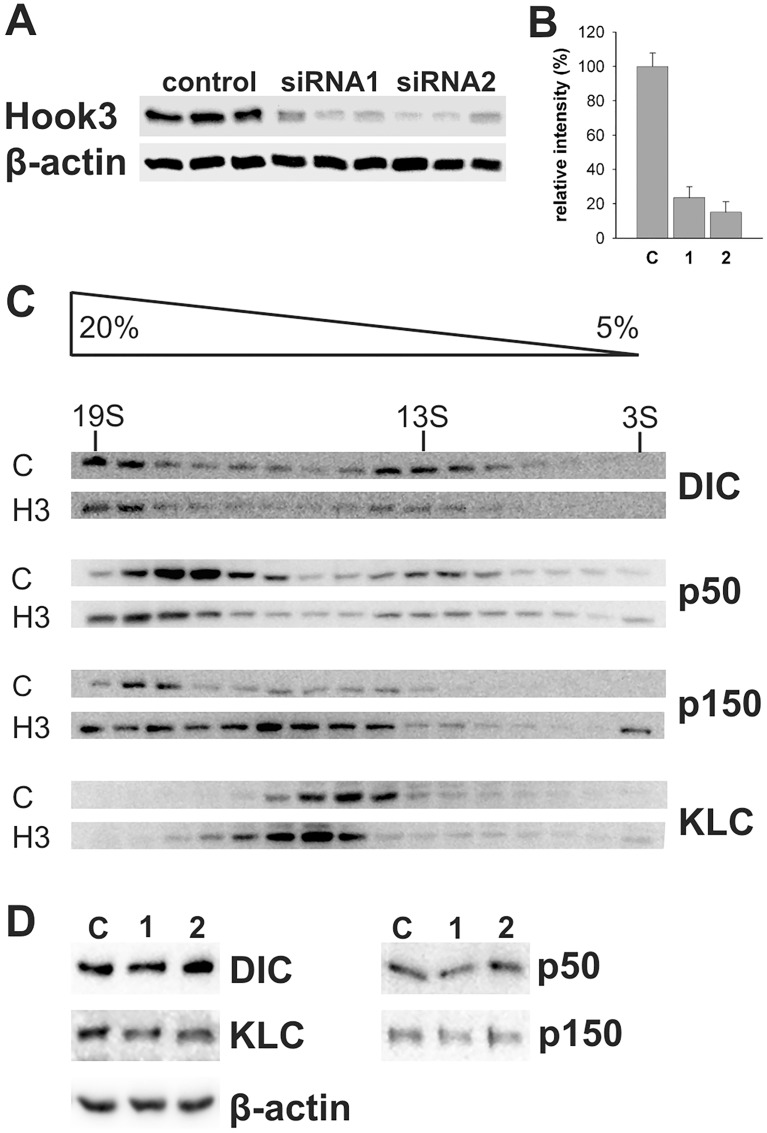
Effect of Hook3 siRNA on cytoplasmic transport complexes. N2A cells were transfected with control siRNA or one out of two Hook3 siRNAs and incubated for 72 h. (a) Knockdown of Hook protein levels. Cells were harvested 72 h after transfection and analyzed by SDS-PAGE and Western blotting. (b) Densitometric analysis of protein immunoreactivity. The averaged Hook3 immunoreactivity of Hook3 siRNA transfected cells is 24% and 15% of control level respectively. C: control RNA, 1: Hook3 siRNA1, 2: Hook3 siRNA2. *C*. Sedimentation analysis. Lysates of N2A cells transfected with control or Hook3 siRNA2 and sedimentation standards thyroglobulin (19S), katalase (13S) and BSA (3S) were subjected to 5–20% sedimentation analysis followed by SDS-PAGE and Western blotting. Knockdown of Hook3 shifts the presence of p150 to a protein complex smaller than dynactin, whereas between control and Hook3 siRNA treated cells no differences in DIC, p50 and KLC distribution were detected. Sucrose gradient and peak position of sedimentation standards are indicated at the top. (d) Western blot analysis of cell lysates transfected with control or Hook3 siRNA. Antibodies to DIC, KLC, p50, p150 and β-actin indicate no alteration in the total amount of these proteins by treatment with Hook3 siRNA2. A similar result was obtained with Hook3 siRNA1.

Retrograde transport is dependent on the dynein-dynactin complex. To test whether reduced Hook3 expression alters the integrity of the dynein-dynactin complex, density gradient sedimentation analysis was performed with N2A cell lysates. Transfection of N2A cells with Hook3 siRNAs leads to a reduction of Hook3 expression to 24% and 15% of control level respectively (Fig. 6a and 6b). Separation of protein complexes by 5–20% sucrose gradient centrifugation and immunoblotting of collected fractions reveals the distribution of dynein intermediate chain (DIC), p50, p150 and kinesin light chain (KLC) in different protein complexes (Fig. 6c). DIC precipitates in a 20S protein complex that indicates an intact dynein structure. We found no shift of DIC to a protein complex of lower molecular weight in cells transfected with Hook3 siRNAs. The same result was obtained for the dynactin protein p50 and KLC. Surprisingly, Hook3 knockdown leads to a shift of p150 to a complex of lower molecular weight than dynactin. This smaller complex that contains p150 and not p50/DIC could be an indication of defective dynactin assembly. To rule out whether a reduction in protein levels of transport proteins is hindering endosomal transport, we tested for the total amounts of DIC, KLC, p50 and p150 in control and H3 siRNA transfected cells. We found no impact of H3 siRNAs treatment on levels of these transport proteins (Fig. 6d). A contribution of Hook3 to dynactin assembly might explain the delay in endosomal transport in cells with downregulated Hook3 (Fig. 5c).

### Reduced Hook3 expression enhances β-amyloid production

**Fig 7:**
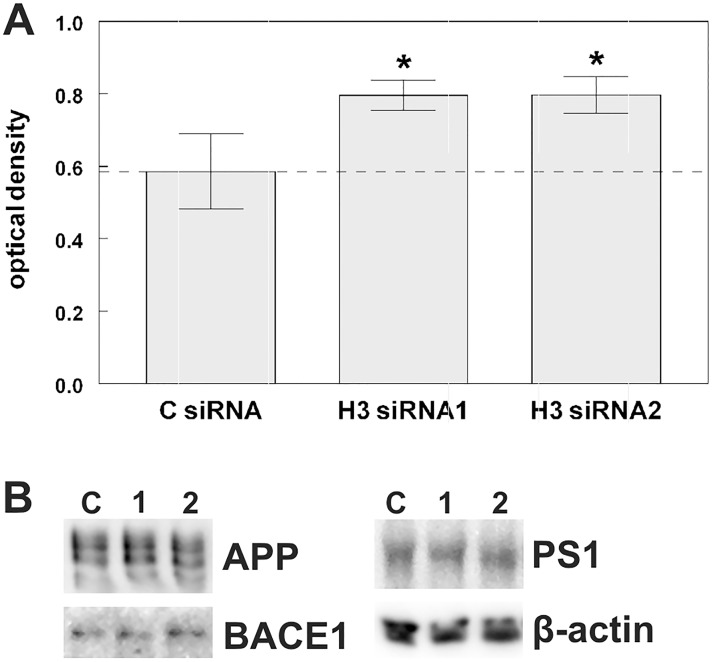
Hook3 knockdown increases production of endogenous β-amyloid in N2A cells. (a) N2A cells were transfected with two different siRNAs targeting Hook3 and with non-target siRNA as control (n = 3). Beta-amyloid accumulated for 48h in cell culture media was quantified by a murine amyloid-specific ELISA. Hook3 downregulation by two different siRNAs causes a 35% increase in secreted β-amyloid. * p < 0.05 compared to control group with student’s t-test. Data are means ± SD. (b) Western blot analysis of cell lysates demonstrate that transfection with Hook3 siRNA does not alter the levels of intracellular APP, BACE1 and PS1.

It has been shown previously that alteration of endosomal trafficking leads to enhanced β-amyloid production [13,14]. To examine if the delayed endosomal transport in cells with Hook3 knockdown alters the processing of APP, we quantified the amount of secreted β-amyloid of siRNA transfected N2A cells by ELISA. Murine N2A cells express APP endogenously, which allows an easy detection of secreted β-amyloid peptides. Downregulation of Hook3 expression induced a significant 36% increase in β-amyloid peptide 1–40 in cell culture media. This was replicated with two different Hook3 siRNAs (Fig. 7a). In cell lysates no alterations in the protein levels of APP, BACE1 and presenilin 1 (PS1) were detected (Fig. 7b) and Hook3 siRNA treatment of COS7 cells transfected with APP-YFP does not induce fluorescent APP accumulation (S9 Fig.). Therefore, enhanced β-amyloid production is not based on a general increase of APP or APP-cleaving enzymes. We propose that the slowing down of endosomal transport caused by reduced Hook3 availability leads to a prolonged residence of APP in early endosomes and to enhanced β-secretase cleavage of APP.

## Discussion

This is the first report of Hook protein expression in human brain tissue, revealing a decrease of Hook1 and Hook3 protein isoforms in AD and its association with tau protein aggregates. Our findings suggest that a lack of functional Hook3 protein in later stages of the disease contributes to the disturbed intracellular transport as observed in AD. As a consequence of Hook3 downregulation in a cell culture model, we detect an increase of amyloidogenic APP processing. Here we speculate that Hook3 protein may play a critical role in pathogenic events exacerbating AD.

### Expression of Hook proteins in human brain without and with tau pathology

The aim of this study was to investigate Hook protein function in relationship to the expression in normal aged brain tissue and in brain tissue of patients affected by AD. Although the Hook protein sequence is rather conserved [16], expression level and cellular localization of the three isoforms in human control brain are diverse. Hook3 is the major isoform in the brain and has the strongest expression detected by quantitative PCR, on Western blots and in immunohistochemistry. The specific neuronal localization is confined to cytoplasm, neurites and nucleus, where Hook3 may serve different functions. Hook1 antiserum yielded a weak parenchymal background staining with clear labeling of axonal varicosities in layers 2 and 3 in the cortex and in the hippocampus, arguing that Hook1 protein is subject to axonal transport and we speculate that it might have a function in presynaptic vesicle transport.

In control brain Hook2 immunohistochemistry gives a weak labeling of small nuclei of unknown origin. The association of Hook2 immunoreactivity with amyloid plaques in AD brain prompted us to investigate its cellular origin. Since β-amyloid and Hook2 immunoreactivities were not overlapping, we identified S100β-positive extensions of reactive astrocytes within of amyloid plaques as a major source of Hook2 expression. This has also been confirmed on reactive astrocytes in tau transgenic P301L mice. Furthermore, a significant increase in Hook2 mRNA expression in AD brain may stem from the widespread astrogliosis.

In brain of AD patients Hook1 and Hook3 antibodies stained neurofibrillary tangles, dystrophic neurites and neuropil threads in various brain regions. Hook1 antibody gave a more distinct labeling of pathological tau aggregates than Hook3 antibody due to less endogenous staining in control brain. What is more interesting than the Hook3 association with tau aggregates is the general decrease of Hook3 immunoreactivity in tangle-bearing and non tangle-bearing neurons in AD brain. In addition to the Hook1 and Hook3 protein sequestration on tau aggregates, which affects only a limited number of neurons, there is a widespread loss of Hook1 and Hook3 immunoreactivity in AD, which consequences were further explored.

Staining of tau deposits with Hook1 and Hook3 antibodies was also observed in tau transgenic P301L mice revealing a consistent association of Hook proteins with aggregated tau. However, in the animal model of tauopathy the striking loss of Hook3 immunoreactivity was not present, pointing to a missing upstream event in this animal model.

### Reduced expression of Hook1 and Hook3 in brain tissue of patients with AD

The decrease in cellular Hook immunoreactivity in AD observed by immunohistochemistry was verified by quantification of Hook proteins in brain homogenates. We used homogenates from the temporal cortex (Brodmann area 22) because in early stages of AD (Braak stages I-III) this area is largely free of neurofibrils, whereas in late AD stages (Braak stages V-VI) the temporal cortex is severely affected by tau pathology [28]. In the cytoplasmic fractions and in the whole homogenates the amount of soluble and of total Hook1 and Hook3 decreases with augmented severity of AD pathology. Hook2 was not detected on Western Blots possibly as a result of low Hook2 mRNA copy numbers. Sequestration of Hook1 and Hook3 to tau deposits in AD is triggered by the presence of tau aggregates with beta-sheet structure but is not affected by tau hyperphosphorylation. However, there are differences between Hook1 and Hook3. The amount of Hook1 strongly varies in control samples. Hook3 is highly abundant in controls and is reduced in all samples from patients in Braak stages V-VI. Thus Hook3 is specifically depleted in the cytoplasm of neurons in AD, which is not caused by a reduced mRNA expression.

The loss of soluble Hook1 and Hook3 protein cannot held responsible solely to the sequestration to tau deposits, because the total amount of both protein is still reduced to 66% and 61%, respectively. A binding of Hook1 and Hook3 to tau oligomers, which are still susceptible to intracellular protein degradation might account for their protein loss. Alternatively, a reduced translation was observed in early and late stages of AD [39,40,41]. It is also likely that the detected reduction in Hook proteins is a combination of changes in translation and an enhanced protein turnover [42,43,44].

### Hook3 depletion enhances β-amyloid production

Knockdown of Hook3 mRNA was performed to investigate the effect of Hook3 depletion on endosomal transport and β-amyloid production. Transfecting HeLa cells with Hook3 siRNA efficiently reduces Hook3 expression and causes a slowdown of centripetal transport and degradation of EGF. Xu *et al*. [23] demonstrated that Hook proteins are associated in a protein complex that interacts with the HOPS complex and that depletion of all three Hook isoforms slows down maturation of late endosomes to lysosomes. Other studies demonstrated a prominent role of Hook3 in vesicular trafficking. Hook3 promotes transport of scavenger receptor A to lysosomes and it is a target of *Salmonella* toxin SpiC [45,46]. Slowing of centripetal transport may be caused by a lack of Hook3 during dynein complex assembly. Density gradient centrifugation reveals that in Hook3 depleted cells the p150 containing protein complex is smaller than dynactin. A reduced availability of intact dynactin might be a reason for impaired transport of late endosomes towards the cell center.

Furthermore, we demonstrated that depletion of Hook3 enhances endogenous β-amyloid generation in N2A cells. Because no alteration in protein levels of APP, BACE1 and PS1 were observed, it is conceivable that a prolonged co-residence of APP and BACE1 in the endosomal compartment leads to enhanced β-amyloid production. The intracellular trafficking pathways of APP and BACE1 are largely segregated starting at the early endosome. Whereas full length APP is rapidly recycled from the plasma membrane and transported to the late endosome/lysosome is BACE1 predominantly transported to the recycling endosome [47]. Downregulation of SorlA or a protein of the retromer complex, that sort APP from early endosomes to the Golgi apparatus, leads to an increase in β-amyloid production in the endosomal-lysosomal compartments and is a risk factor for late onset AD [48,49,50]. Recent research shows that Hook proteins may act as scaffolding protein promoting the interaction of cargo with molecular motors and modulating the motility by affecting cargo-bound kinesin or dynein processivity [51]. By affecting cargo-motor protein attachment [52] and motor processivity [53] cargo can be re-routed and APP/BACE1 convergence can be increased in time and location. Thus we propose that the slowdown of vesicular trafficking by Hook3 deficiency enhances β-amyloid generation through the prolonged interaction between APP and BACE1 and increased encounter in the endosomal compartment.

In summary, we demonstrated that expression of Hook1 and Hook3 is reduced in AD. This reduction of soluble Hook proteins is triggered by a sequestration to tau aggregates. Because depletion of Hook3 slows down endosomal transport and may affect endosomal cargo routing it increases β-amyloid generation and contributes to the dysfunction of endosomal-lysosomal compartments in later stages of AD. Future endeavors revealing the exact role of Hook3 in endosomal trafficking might enlighten its role in the pathogenesis of AD.

## Supporting Information

S1 Fig Low magnification overview of the hippocampal CA3 formation. This figure serves as a supplement to Fig. 1D of Hook1 and Hook3 immunoreactivity in controls and AD brains. Due to the low power pathological structures and normal labeling cannot be discriminated. Scale bar = 500 μm.
(TIF)

S2 Fig Hook1 localization in controls and AD brains. Immunohistochemical staining of the temporal, the frontal and the occipital cortex with anti-Hook1 antibody. Hook1 antibody labels small nuclei probably of oligodendroglial origin (arrowhead) and axonal swellings (arrow) present in cortical layer I to layer III in the temporal and the frontal cortex of control brain. Only few axonal swellings are observed in the occipital cortex. In AD brain the parenchymal background staining and the axonal labeling are diminished. Neurofibrillary tangles (asterisk) and dystrophic neuritis (+) associated with β-amyloid deposits are strongly marked by Hook1 antibody. Scale bar = 25 μm.
(TIF)

S3 Fig Hook2 localization in controls and AD brains. Immunohistochemical staining of the temporal, the frontal and the occipital cortex with anti-Hook2 antibody. Hook2 antibody labels the endothelial part of blood vessels (x) and nuclei of small cells (arrowhead) in control brain. In AD brain amyloid plaques of different size (+) are detected, in which immunoreactivity is mostly associated with granular or filamentous structures (occipital cortex) within the amyloid deposits. This filamentous immunoreactivity is not associated with dystrophic neurites and is pronounced in amyloid deposits in the occipital cortex. Scale bar = 25 μm.
(TIF)

S4 Fig Hook3 localization in controls and AD brains. Immunohistochemical staining of the temporal, the frontal and the occipital cortex with anti-Hook3 antibody. Incubation of control brain slices with Hook3 antibody reveals a strong neuronal staining especially of pyramidal cells (arrowhead). Immunoreactivity is present in the nucleus with a speckled appearance and with a granular distribution within the cell body and proximal extensions. The neuronal immunoreactivity is strongly reduced in cortical areas of AD brain and dystrophic neurites in neuritic plaques (+), neurofibrillary tangles (asterisk) and neuropil threads (arrow) are detected. Scale bar = 25 μm.
(TIF)

S5 Fig Astrocytic localization of Hook2 revealed by co-labeling with anti-S100β or anti-GFAP in human brain. (a) In AD the frontal cortex immunoreactivity of Hook2 is largely confined to fine astrocytic processes pervading the amyloid plaques (+) as detected with monoclonal S100β antibody. In addition, activated astrocytes in the vicinity of amyloid plaques encasing blood vessels are also Hook2 positive (x). Therefore, amyloid plaque detection by the Hook2 antibody is mainly due to reactivity with astrocytic subcompartments within the amyloid deposits. (b) In control brain Hook2 labeling is occasionally found in stellate astrocytes in the temporal cortex as confirmed by GFAP colocalization. GFAP-positive astrocytic end-feet encircling blood vessels depicted in the upper left corner are also weakly positive for Hook2 (x). Scale bar = 10 μm.
(TIF)

S6 Fig Western blot analysis of the cytoplasmic fractions of brain homogenates. Grey substance of Brodmann area 22 (temporal cortex) was homogenized in detergent-free buffer and was subsequently ultracentrifuged. Twenty-three μg of protein of each supernatant were loaded on SDS-PAGE. (a) Labeling with Hook1 antibody detects the protein band of Hook1 at 85 kDa (arrow). In addition, another protein of higher molecular weight is unspecifically detected. (b) No 83 kDa Hook2 (arrow) is detected by Hook2 antibody. At lower molecular weight possibly degradation products are visible. (c) Hook3 antibody labeling only marks a Hook3 band at 83 kDa (arrow).
(TIF)

S7 Fig Schematic of Alexa Fluor 555-EGF fluorescence intensity measurements. (a) Endocytotic uptake of Alexa Fluor 555-EGF was quantified by calculating the mean fluorescence intensity measured as a fluorescence profile across each cell (see white line). Transfection of dominant-negative dynamin 1 (Dyn1dn) completely blocked EGF uptake, whereas Hook3 siRNA (H3 siRNA) treatment induced loss of perinuclear clustering 90 min after EGF application, but did not change overall EGF uptake. (b) Fluorescence intensity profile of labeled EGF in HeLa cells. Quantification of non-homogenous EGF distribution in control siRNA (C siRNA) treated cells compared to Hook3 siRNA treated cells was achieved by calculating the difference of maximum fluorescence intensity averaged in a 1.7 μm region and averaged minimum fluorescence intensity. Exemplary regions used for calculating minimum and maximum are depicted in (a) by a thick white line intercept and the difference is marked in case of a H3 siRNA treated cell in diagram (b).
(TIF)

S8 Fig Hook isoform expression in human brain. A boxplot diagram showing the variations in Hook isoform mRNA levels between controls and Alzheimer disease patients. The line across the box represents the median while the limits of the box represent lower quartile (25th percentile), upper quartile (75th) and the whiskers with smallest observation as well as the largest observations are shown. Hook2 mRNA is significantly increased in AD (Braak stage V-VI), whereas Hook1 mRNA level remain constant and Hook3 mRNA shows an insignificant trend of decrease in AD. A Mann-Whitney-U-test was used for statistical evaluation. Asterisk indicates statistically significant difference (**p < 0.01)
(TIF)

S9 Fig Endosomal localization of APP-YFP in COS7 cell 72h after control siRNA or Hook3 siRNA2 treatment. Comparable APP-YFP fluorescence can be detected in control and Hook3 siRNA2 treated cells. Scale bar = 20 μm.
(TIF)
