# Supplementary figures and images for: Hook Proteins: Association with Alzheimer Pathology and Regulatory Role of Hook3 in Amyloid Beta Generation
Source: PLoS One. 2015 Mar 23;10(3):e0119423. doi: 10.1371/journal.pone.0119423 (PMC4370497; doi:10.1371/journal.pone.0119423)

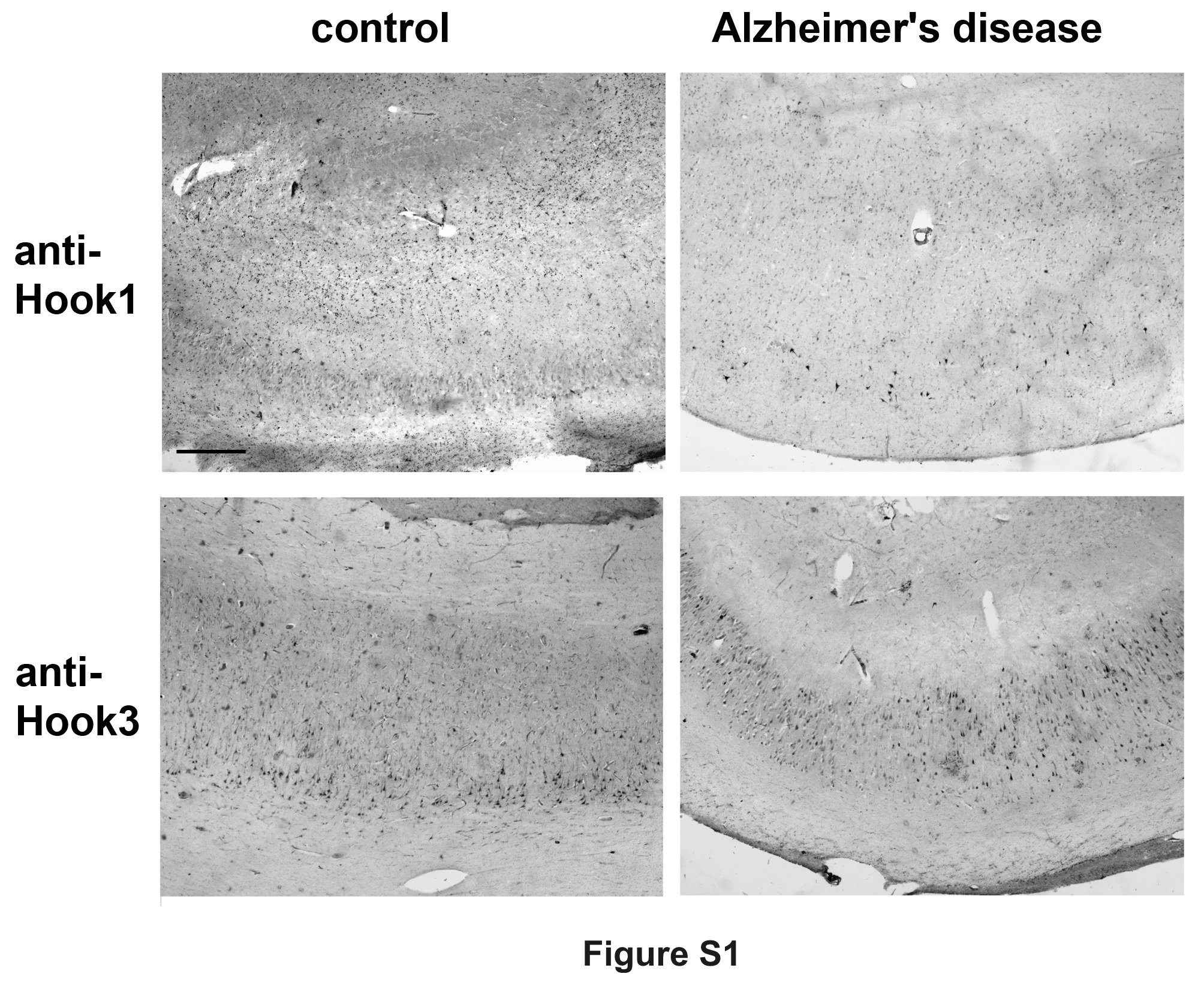

Supplement: S1 Fig — This figure serves as a supplement to Fig. 1D of Hook1 and Hook3 immunoreactivity in controls and AD brains. Due to the low power pathological structures and normal labeling cannot be discriminated. Scale bar = 500 μm. (TIF) [file pone.0119423.s001.tif]

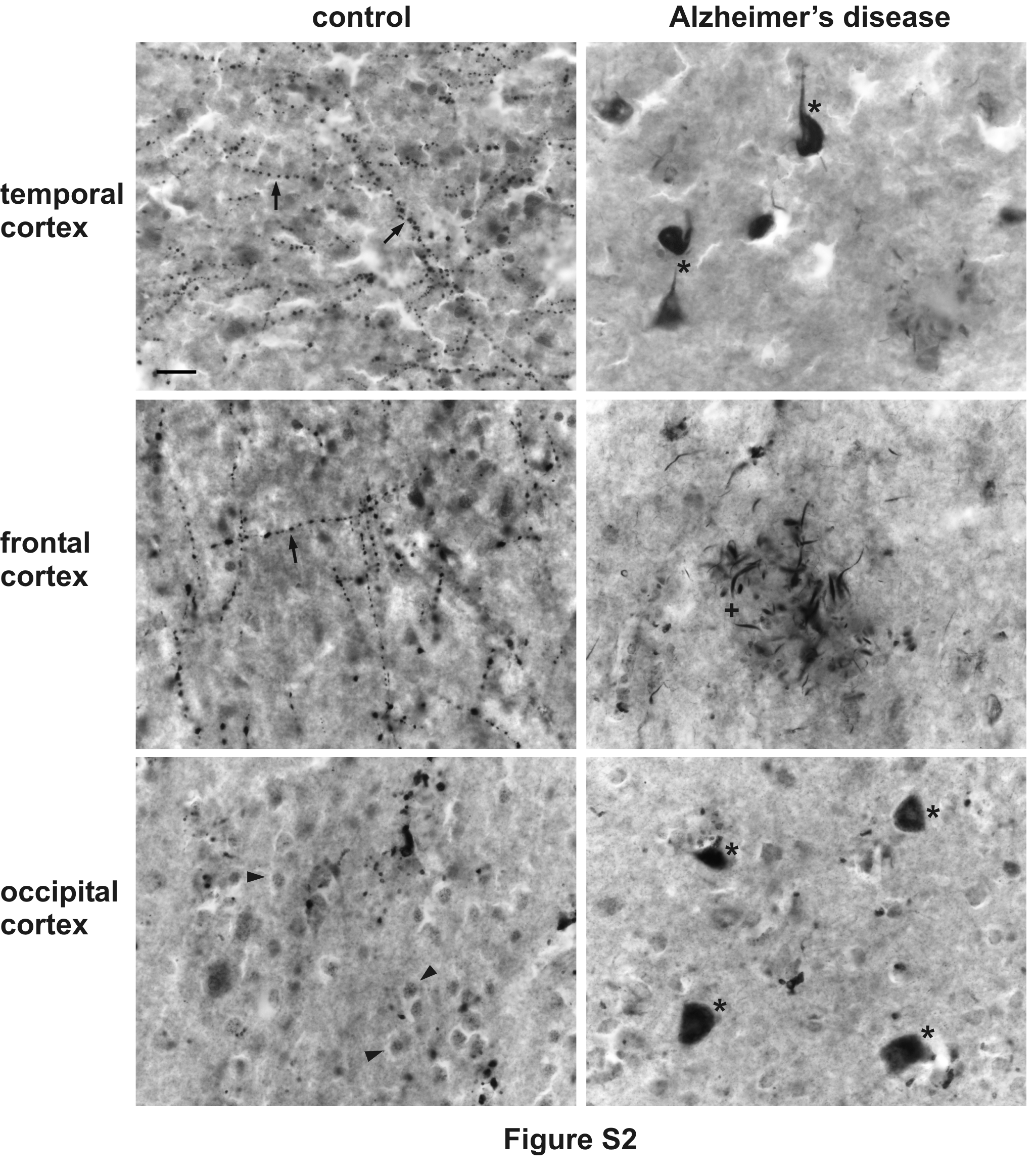

Supplement: S2 Fig — Immunohistochemical staining of the temporal, the frontal and the occipital cortex with anti-Hook1 antibody. Hook1 antibody labels small nuclei probably of oligodendroglial origin (arrowhead) and axonal swellings (arrow) present in cortical layer I to layer III in the temporal and the frontal cortex of control brain. Only few axonal swellings are observed in the occipital cortex. In AD brain the parenchymal background staining and the axonal labeling are diminished. Neurofibrillary tangles (asterisk) and dystrophic neuritis (+) associated with β-amyloid deposits are strongly marked by Hook1 antibody. Scale bar = 25 μm. (TIF) [file pone.0119423.s002.tif]

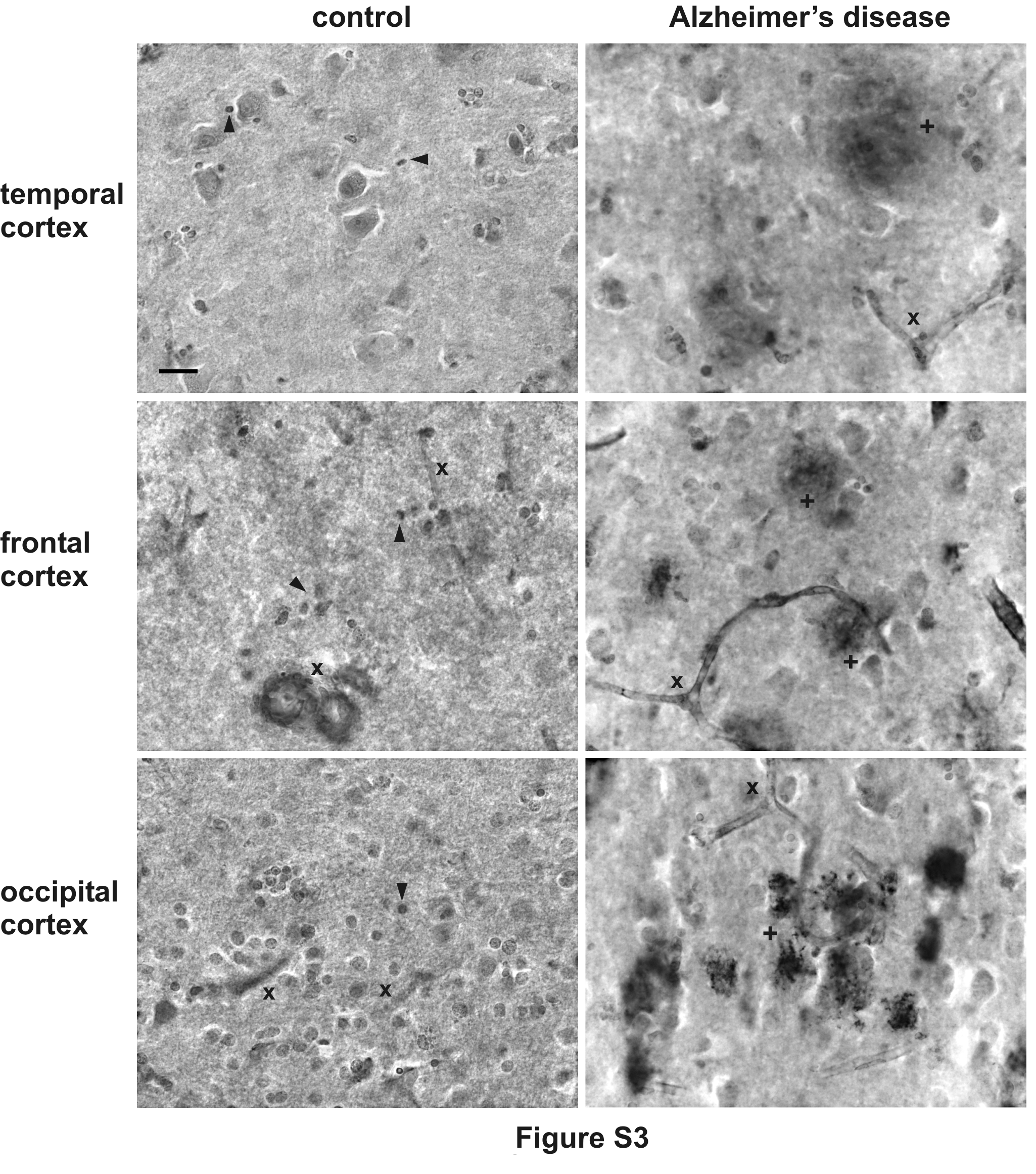

Supplement: S3 Fig — Immunohistochemical staining of the temporal, the frontal and the occipital cortex with anti-Hook2 antibody. Hook2 antibody labels the endothelial part of blood vessels (x) and nuclei of small cells (arrowhead) in control brain. In AD brain amyloid plaques of different size (+) are detected, in which immunoreactivity is mostly associated with granular or filamentous structures (occipital cortex) within the amyloid deposits. This filamentous immunoreactivity is not associated with dystrophic neurites and is pronounced in amyloid deposits in the occipital cortex. Scale bar = 25 μm. (TIF) [file pone.0119423.s003.tif]

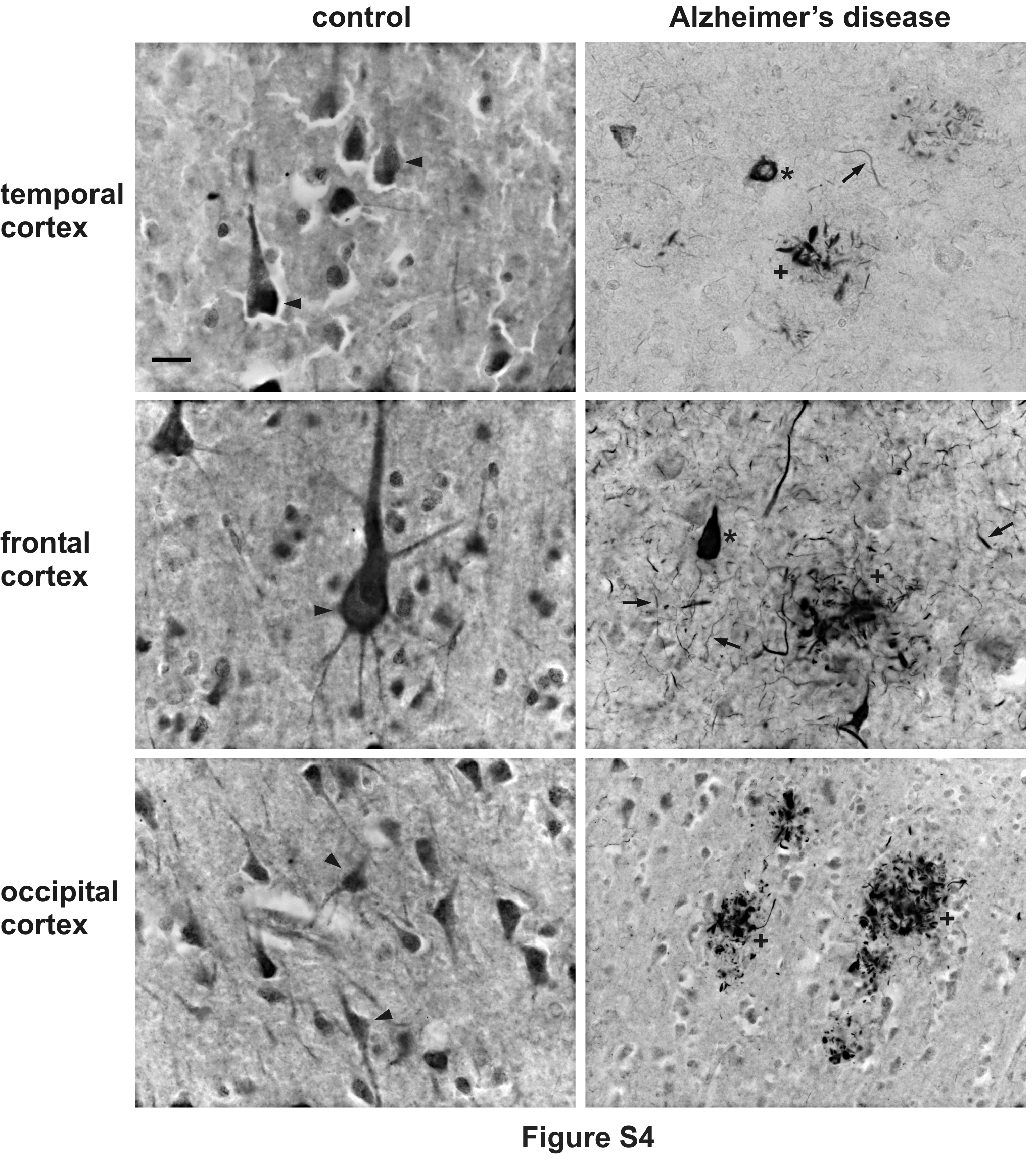

Supplement: S4 Fig — Immunohistochemical staining of the temporal, the frontal and the occipital cortex with anti-Hook3 antibody. Incubation of control brain slices with Hook3 antibody reveals a strong neuronal staining especially of pyramidal cells (arrowhead). Immunoreactivity is present in the nucleus with a speckled appearance and with a granular distribution within the cell body and proximal extensions. The neuronal immunoreactivity is strongly reduced in cortical areas of AD brain and dystrophic neurites in neuritic plaques (+), neurofibrillary tangles (asterisk) and neuropil threads (arrow) are detected. Scale bar = 25 μm. (TIF) [file pone.0119423.s004.tif]

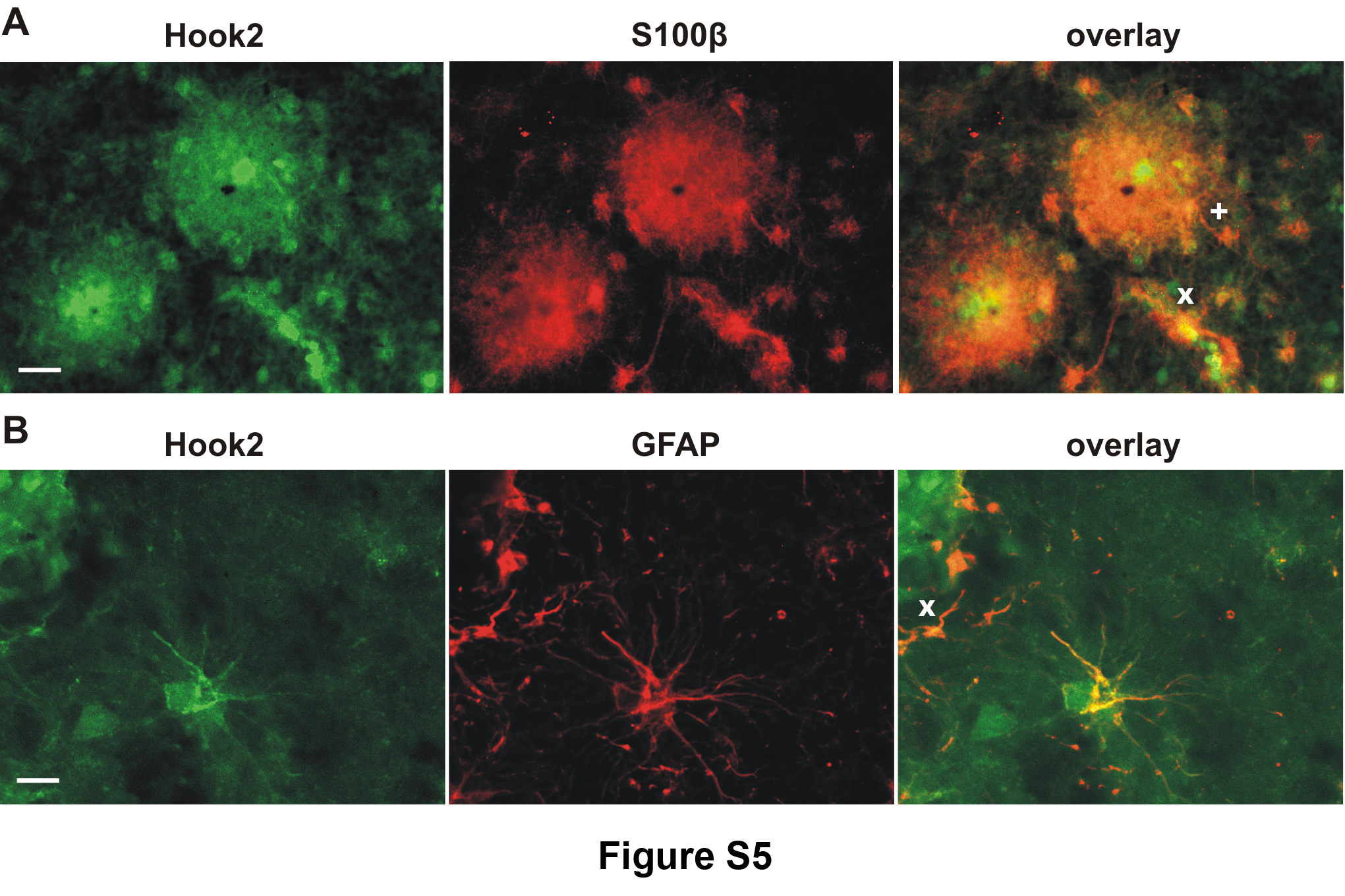

Supplement: S5 Fig — (a) In AD the frontal cortex immunoreactivity of Hook2 is largely confined to fine astrocytic processes pervading the amyloid plaques (+) as detected with monoclonal S100β antibody. In addition, activated astrocytes in the vicinity of amyloid plaques encasing blood vessels are also Hook2 positive (x). Therefore, amyloid plaque detection by the Hook2 antibody is mainly due to reactivity with astrocytic subcompartments within the amyloid deposits. (b) In control brain Hook2 labeling is occasionally found in stellate astrocytes in the temporal cortex as confirmed by GFAP colocalization. GFAP-positive astrocytic end-feet encircling blood vessels depicted in the upper left corner are also weakly positive for Hook2 (x). Scale bar = 10 μm. (TIF) [file pone.0119423.s005.tif]

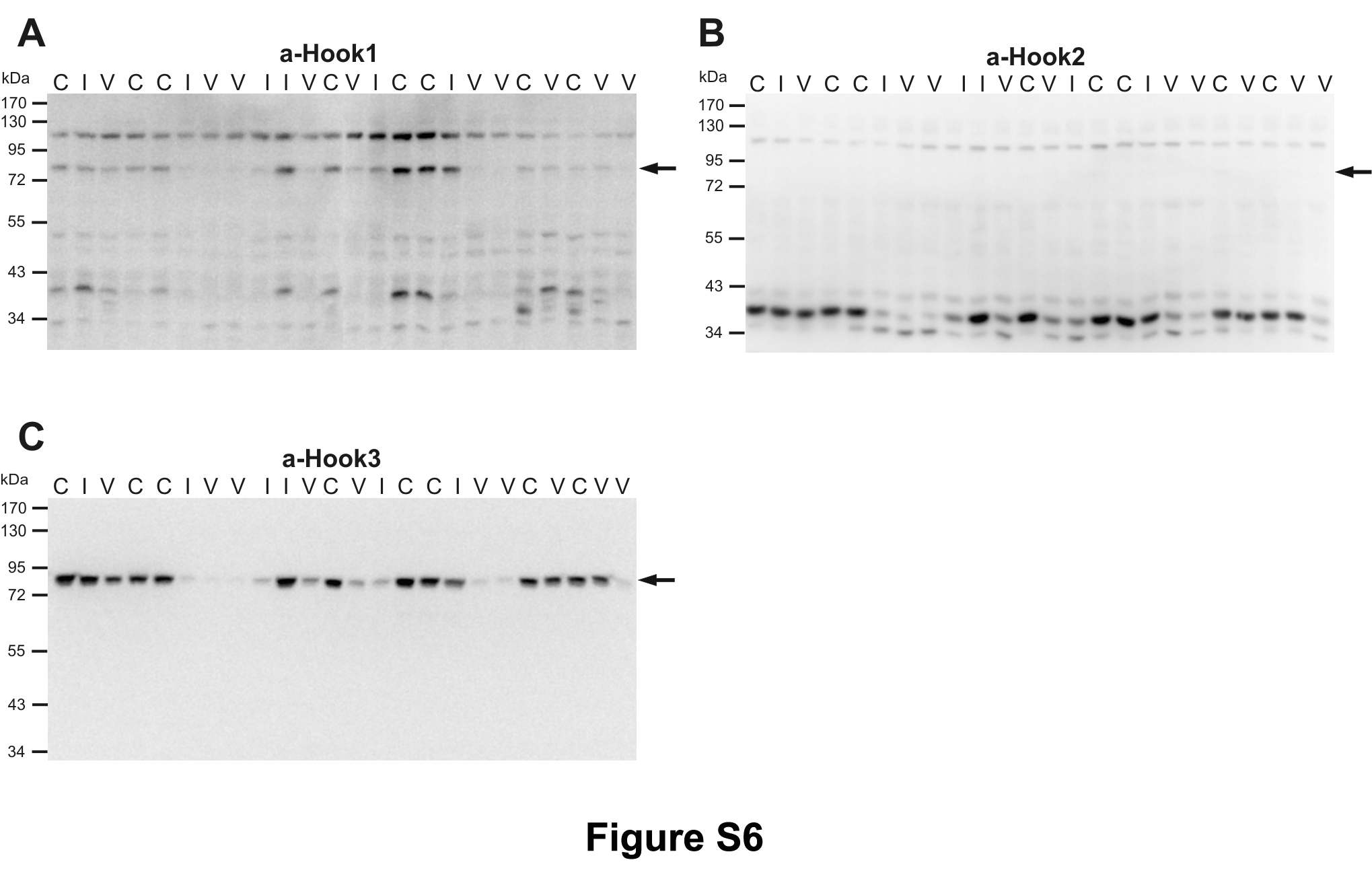

Supplement: S6 Fig — Grey substance of Brodmann area 22 (temporal cortex) was homogenized in detergent-free buffer and was subsequently ultracentrifuged. Twenty-three μg of protein of each supernatant were loaded on SDS-PAGE. (a) Labeling with Hook1 antibody detects the protein band of Hook1 at 85 kDa (arrow). In addition, another protein of higher molecular weight is unspecifically detected. (b) No 83 kDa Hook2 (arrow) is detected by Hook2 antibody. At lower molecular weight possibly degradation products are visible. (c) Hook3 antibody labeling only marks a Hook3 band at 83 kDa (arrow). (TIF) [file pone.0119423.s006.tif]

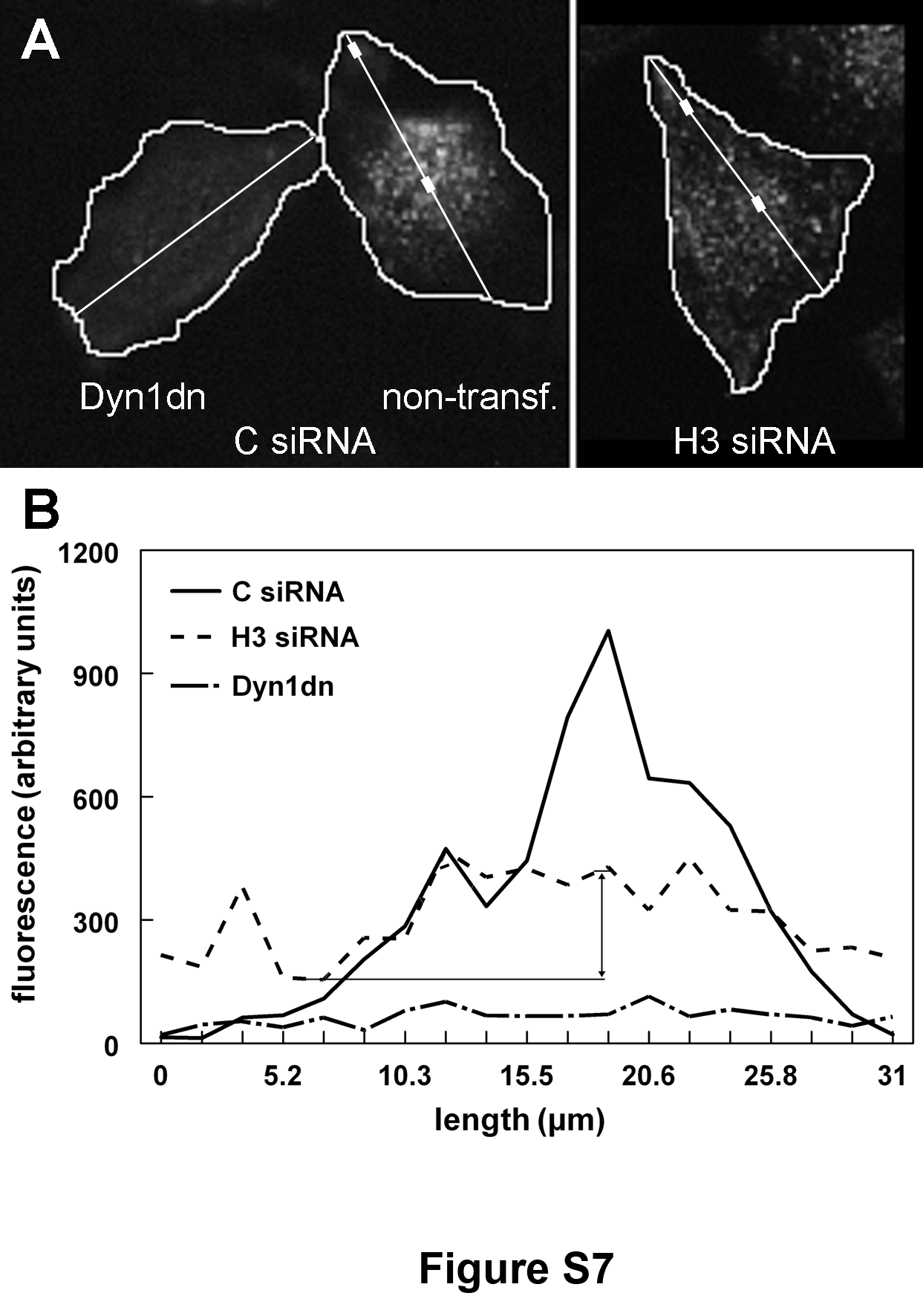

Supplement: S7 Fig — (a) Endocytotic uptake of Alexa Fluor 555-EGF was quantified by calculating the mean fluorescence intensity measured as a fluorescence profile across each cell (see white line). Transfection of dominant-negative dynamin 1 (Dyn1dn) completely blocked EGF uptake, whereas Hook3 siRNA (H3 siRNA) treatment induced loss of perinuclear clustering 90 min after EGF application, but did not change overall EGF uptake. (b) Fluorescence intensity profile of labeled EGF in HeLa cells. Quantification of non-homogenous EGF distribution in control siRNA (C siRNA) treated cells compared to Hook3 siRNA treated cells was achieved by calculating the difference of maximum fluorescence intensity averaged in a 1.7 μm region and averaged minimum fluorescence intensity. Exemplary regions used for calculating minimum and maximum are depicted in (a) by a thick white line intercept and the difference is marked in case of a H3 siRNA treated cell in diagram (b). (TIF) [file pone.0119423.s007.tif]

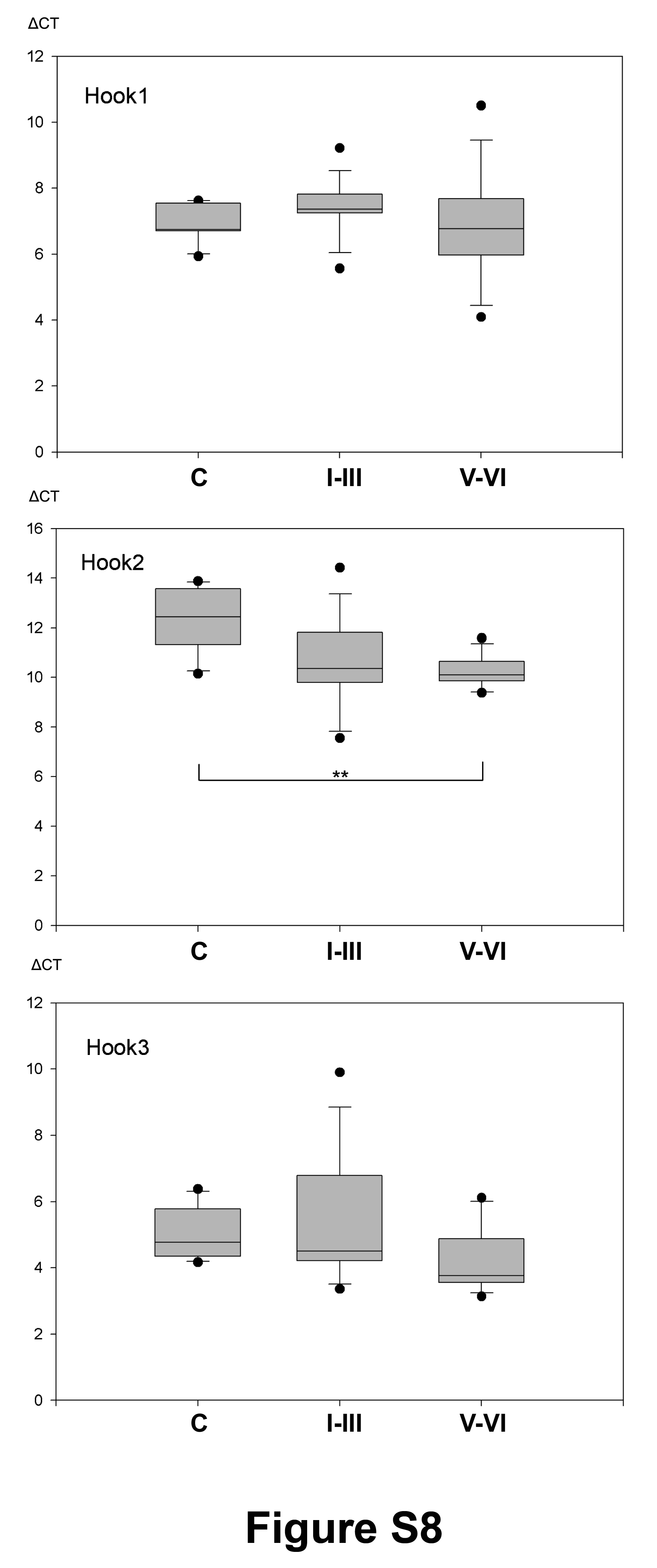

Supplement: S8 Fig — A boxplot diagram showing the variations in Hook isoform mRNA levels between controls and Alzheimer disease patients. The line across the box represents the median while the limits of the box represent lower quartile (25th percentile), upper quartile (75th) and the whiskers with smallest observation as well as the largest observations are shown. Hook2 mRNA is significantly increased in AD (Braak stage V-VI), whereas Hook1 mRNA level remain constant and Hook3 mRNA shows an insignificant trend of decrease in AD. A Mann-Whitney-U-test was used for statistical evaluation. Asterisk indicates statistically significant difference (**p < 0.01) (TIF) [file pone.0119423.s008.tif]

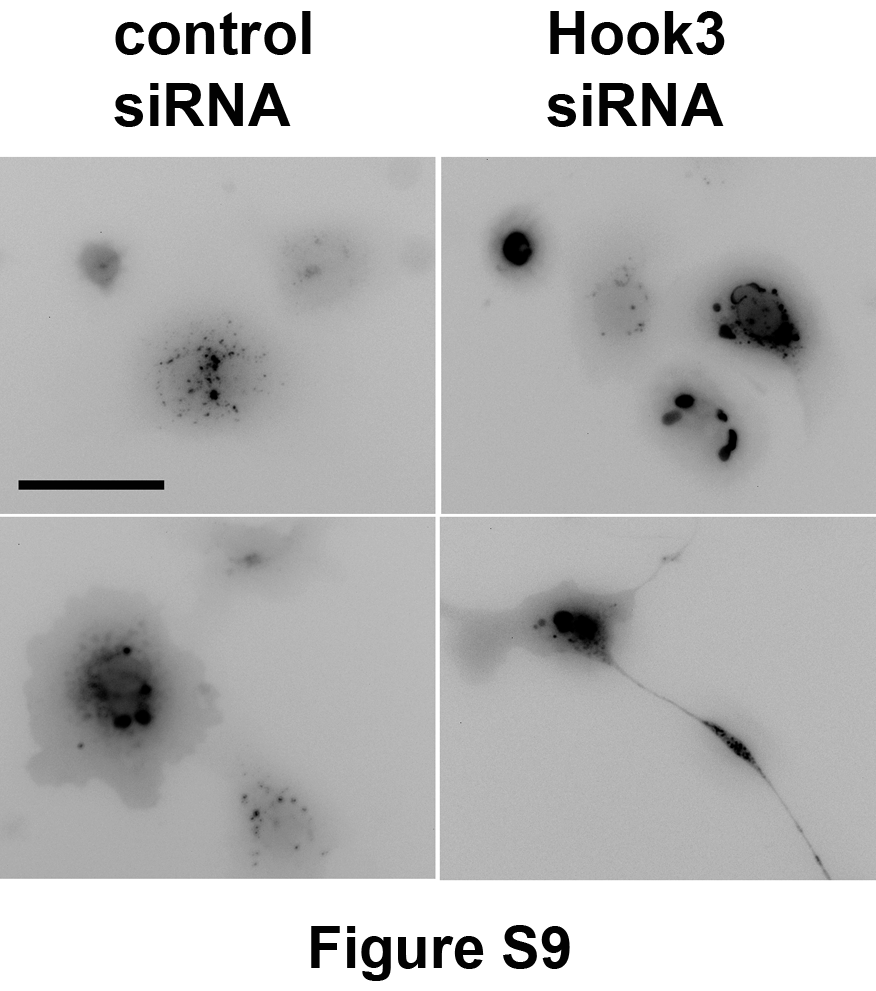

Supplement: S9 Fig — Comparable APP-YFP fluorescence can be detected in control and Hook3 siRNA2 treated cells. Scale bar = 20 μm. (TIF) [file pone.0119423.s009.tif]
